# Differential mechanisms of posterior cingulate cortex downregulation and symptom decreases in posttraumatic stress disorder and healthy individuals using real‐time fMRI neurofeedback

**DOI:** 10.1002/brb3.2441

**Published:** 2021-12-18

**Authors:** Andrew A. Nicholson, Daniela Rabellino, Maria Densmore, Paul A. Frewen, David Steryl, Frank Scharnowski, Jean Théberge, Richard W.J. Neufeld, Christian Schmahl, Rakesh Jetly, Ruth A. Lanius

**Affiliations:** ^1^ Department of Psychiatry and Behavioural Neurosciences McMaster University Hamilton Ontario Canada; ^2^ Department of Cognition, Emotion, and Methods in Psychology University of Vienna Vienna Austria; ^3^ Department of Neuroscience, Western University London Ontario Canada; ^4^ Imaging Lawson Health Research Institute London Ontario Canada; ^5^ Department of Psychology, Western University London Ontario Canada; ^6^ Department of Medical Biophysics Western University London Ontario Canada; ^7^ Department of Psychiatry, Western University London Ontario Canada; ^8^ Department of Diagnostic Imaging St. Joseph's Healthcare London Ontario Canada; ^9^ Department of Psychology University of British Columbia, Okanagan Kelowna British Columbia Canada; ^10^ Department of Psychosomatic Medicine and Psychotherapy, Central Institute of Mental Health Mannheim Heidelberg University Heidelberg Germany; ^11^ Canadian Forces Health Services Ottawa Ontario Canada

**Keywords:** machine learning, neurofeedback, post‐traumatic stress disorder, real‐time fMRI

## Abstract

**Background:**

Intrinsic connectivity networks, including the default mode network (DMN), are frequently disrupted in individuals with posttraumatic stress disorder (PTSD). The posterior cingulate cortex (PCC) is the main hub of the posterior DMN, where the therapeutic regulation of this region with real‐time fMRI neurofeedback (NFB) has yet to be explored.

**Methods:**

We investigated PCC downregulation while processing trauma/stressful words over 3 NFB training runs and a transfer run without NFB (total *n* = 29, PTSD *n* = 14, healthy controls *n* = 15). We also examined the predictive accuracy of machine learning models in classifying PTSD versus healthy controls during NFB training.

**Results:**

Both the PTSD and healthy control groups demonstrated reduced reliving symptoms in response to trauma/stressful stimuli, where the PTSD group additionally showed reduced symptoms of distress. We found that both groups were able to downregulate the PCC with similar success over NFB training and in the transfer run, although downregulation was associated with unique within‐group *decreases* in activation within the bilateral dmPFC, bilateral postcentral gyrus, right amygdala/hippocampus, cingulate cortex, and bilateral temporal pole/gyri. By contrast, downregulation was associated with *increased* activation in the right dlPFC among healthy controls as compared to PTSD. During PCC downregulation, right dlPFC activation was negatively correlated to PTSD symptom severity scores and difficulties in emotion regulation. Finally, machine learning algorithms were able to classify PTSD versus healthy participants based on brain activation during NFB training with 80% accuracy.

**Conclusions:**

This is the first study to investigate PCC downregulation with real‐time fMRI NFB in both PTSD and healthy controls. Our results reveal acute decreases in symptoms over training and provide converging evidence for EEG‐NFB targeting brain networks linked to the PCC.

## Graphical abstract

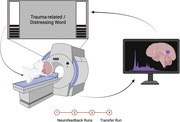

## 1 INTRODUCTION

The advent of functional magnetic resonance imaging (fMRI) has led to unprecedented insights into understanding the neurobiology of posttraumatic stress disorder (PTSD). It has been well documented that PTSD is associated with multiple functional disruptions in the brain that appear to underscore unique symptom presentations of the disorder (Fenster et al., 2018). Real‐time fMRI neurofeedback (rt‐fMRI‐NFB) allows for such neural disruptions to be noninvasively regulated; as such rt‐fMRI‐NFB has been implemented in a broad range of prevalent psychiatric conditions (Linden et al., 2012; Kirsch et al., 2013; Li et al., 2013; Schoenberg and David, 2014; Paret et al., 2019, 2016a; Young et al., 2017; Mehler et al., 2018), including PTSD (Gerin et al., 2016; Nicholson et al., 2016a, 2018; Zotev et al., 2018; Zweerings et al., 2018; Chiba et al., 2019; Misaki et al., 2019; Weaver et al., 2020). Neurobiologically informed treatment interventions are particularly in demand for PTSD as suboptimal response rates to psychotherapy and pharmacological interventions have been reported (Bradley et al., 2005; Stein et al., 2006; Ravindran and Stein, 2009; Haagen et al., 2015; Krystal et al., 2017), where dropout rates remain high, particularly during trauma‐focused interventions (Bisson et al., 2013; Goetter et al., 2015; Kehle‐Forbes et al., 2016; Lewis et al., 2020).

In response to this demand, emerging scientific evidence suggests that directly regulating specific brain areas associated with PTSD symptomatology may be a viable treatment option for those affected by this illness (Reiter et al., 2016; Van der Kolk et al., 2016; Panisch & Hai, 2018; Chiba et al., 2019; Nicholson et al., 2020b, 2020c; Rogel et al., 2020). It has been hypothesized that normalizing the neural circuitry within large scale intrinsic connectivity networks (ICNs) is an essential treatment avenue for reducing PTSD symptoms (Lanius et al., 2015; Koek et al., 2019; Szeszko & Yehuda, 2019; Nicholson et al., 2020a, 2020b; Sheynin et al., 2020). Default mode network (DMN) functional disruptions among individuals with PTSD are thought to be related to traumatic/negative autobiographical memories, distorted and dysregulated self‐referential processing, and alterations in social cognition (Bluhm et al., 2009; Daniels et al., 2010; Lanius et al., 2015; Tursich et al., 2015; Akiki et al., 2017; Fenster et al., 2018; Hinojosa et al., 2019; Frewen et al., 2020; Terpou et al., 2020). Indeed, to suffer from PTSD can be described as living with a disrupted self‐narrative (Gerge, 2020; Lanius et al., 2020), where among individuals with PTSD, especially with early childhood maltreatment, there typically exists a highly rudimentary or shattered sense‐of‐self (Lanius et al., 2020).

The posterior cingulate cortex (PCC) is the major hub of the posterior default mode network (DMN) (Greicius et al., 2003; Buckner et al., 2008; Spreng et al., 2008; Qin and Northoff, 2011; Akiki et al., 2018). The PCC and the DMN are highly associated with PTSD symptoms, and display disrupted functional connectivity both at rest (Bluhm et al., 2009; Sripada et al., 2012; Chen & Etkin, 2013; Tursich et al., 2015; Yehuda et al., 2015; Lanius et al., 2015; Koch et al., 2016; Akiki et al., 2018, 2017; Barredo et al., 2018; Hinojosa et al., 2019; Nicholson et al., 2020a) and during executive functioning tasks in PTSD (Daniels et al., 2010; Melara et al., 2018). During rest, it has been shown previously using graph theoretical analyses that connectivity within the posterior community of the DMN involving the PCC and precuneus may be increased, relative to decreased connectivity within the anterior community of the DMN involving the medial prefrontal cortex (mPFC) (Shang et al., 2014; Kennis et al., 2016; Akiki et al., 2018; Holmes et al., 2018). Additionally, studies exploring seed‐based functional connectivity patterns within the DMN at rest have revealed decreased coupling between the PCC, vmPFC, and other DMN structures, which together have been associated with PTSD symptoms (Bluhm et al., 2009; Qin et al., 2012; Sripada et al., 2012; Koch et al., 2016; Miller et al., 2017; DiGangi et al., 2016). During working memory tasks that require executive functioning, enhanced connectivity of the PCC with other DMN areas has also been reported among individuals with PTSD as compared to increased central executive network (CEN) and salience network (SN) connectivity among healthy individuals (Daniels et al., 2010). With respect to executive functioning tasks in PTSD, suboptimal downregulation of DMN regions may underscore difficulties in disengaging from internally focused self‐referential processing in order to attend to external cognitive demands (Aupperle et al., 2016). Notably, the DMN also exhibits altered activation patterns during threatful‐ and trauma‐related conditions in PTSD. Indeed, a recent meta‐analysis has shown that both reexperiencing and retrieval of trauma‐related autobiographical memories are associated with enhanced activation within the PCC and other DMN regions among individuals with PTSD as compared to healthy controls (Thome et al., 2019). Meta‐analytic results reported elsewhere also suggest that traumatic imagery tasks uniquely induce activation in the PCC, with coactivation of the precuneus, relative to healthy controls (Ramage et al., 2013). Similarly, the presentation of trauma‐versus‐neutral words has been shown to increase activation in the PCC, the mPFC, the midbrain, and the bed‐nucleus of the stria terminalis (BNST), with concomitant decreases in activation within dlPFC emotion regulation areas in PTSD as compared to healthy controls (Awasthi et al., 2020). This is supported by years of experimental work in the field linking these neural correlates with PTSD symptoms during both script‐driven imagery and the recall of trauma‐related autobiographical memories in PTSD (Hopper et al., 2007a; Lanius et al., 2007; Frewen et al., 2011; Mickleborough et al., 2011; Ramage et al., 2013; Liberzon & Abelson, 2016; Fenster et al., 2018; Thome et al., 2019). As such, during trauma‐related stimulus exposure, it has been suggested that enhanced DMN recruitment in PTSD may coincide with self‐related processes that are seemingly fused with experiences of trauma, indeed reflecting the self‐coupled nature of the disorder (Terpou et al., 2019; Lanius et al., 2020). Furthermore, the PCC has been shown to be hyperactive in PTSD during emotion‐processing tasks in comparison to healthy individuals, where critically, longitudinal improvements in PTSD symptoms in response to trauma‐focused cognitive behavioral therapy (CBT) have been found to be associated with decreased PCC activation in youth with PTSD (Garrett et al., 2019). Taken together, regulating the PCC and the DMN may represent a critical avenue to explore with respect to neurobiologically informed treatment interventions for PTSD (Lanius et al., 2015; Akiki et al., 2018; Nicholson et al., 2020c).

In support of this, previous studies in PTSD using electroencephalography neurofeedback (EEG‐NFB), including a randomized controlled trial by our group (Nicholson et al., 2020b), have examined the regulation of brain oscillations tied to the PCC and DMN (Kluetsch et al., 2014; Nicholson et al., 2016b). Notably, one session of EEG‐NFB has been shown to lead to acute decreases in arousal symptoms among individuals with PTSD, which has been associated with a normalization of both DMN and amygdala resting‐state functional connectivity (Kluetsch et al., 2014; Nicholson et al., 2016b). In these aforementioned EEG‐NFB studies, the target of NFB was the desynchronization of alpha rhythms over the PCC. Alpha oscillations are correlated with DMN activation (Mantini et al., 2007; Jann et al., 2009; Clancy et al., 2020), where alpha‐rhythm reductions are commonly observed during the resting‐state in PTSD over the main hubs of the DMN (PCC and mPFC) (Clancy et al., 2020), hypothesized to be related to chronic hyperarousal (Ros et al., 2014; Liberzon & Abelson, 2016; Abdallah et al., 2017; Clancy et al., 2017, 2020; Sitaram et al., 2017; Nicholson et al., 2020c). Additionally, during a 20‐week randomized controlled trial of alpha‐desynchronizing EEG‐NFB in PTSD (Nicholson et al., 2020b), individuals in the experimental group demonstrated significantly reduced PTSD severity scores post‐NFB and at the 3‐month follow‐up, which was associated with a shift towards normalization of DMN resting‐state functional connectivity. Specifically, PTSD patients in the experimental group were found to display decreased PCC connectivity with the anterior DMN after NFB treatment (Nicholson et al., 2020b). It was hypothesized that this may reflect normalized connectivity within over utilized posterior DMN communities consisting of the PCC and precuneus (Akiki et al., 2018; Holmes et al., 2018) after NFB treatment (Nicholson et al., 2020b). Notably, PTSD remission rates as well as decreases in PTSD severity scores in the experimental group were comparable to that of current gold‐standard treatments for PTSD (Nicholson et al., 2020b). Collectively, preliminary results from our previous alpha‐desynchronizing EEG‐NFB studies suggest that feedback signals tied to the DMN, and more specifically the PCC, may represent a viable target for NFB treatment in PTSD. Critically, in comparison to EEG‐NFB, rt‐fMRI‐NFB allows for increased spatial specificity with respect to precisely targeting areas in the brain and provides increased spatial resolution for examining mechanistic evidence associated with regulation.

Recently, the application of rt‐fMRI‐NFB in PTSD has expanded greatly, where previous studies have largely focused on the regulation of the amygdala (Gerin et al., 2016; Nicholson et al., 2016a, 2018; Misaki et al., 2018a, 2018b, 2019; Zotev et al., 2018; Chiba et al., 2019), a limbic region associated with emotion reactivity and highly implicated in PTSD symptoms (Fenster et al., 2018). Nicholson et al. (2016a) found that downregulating the amygdala in PTSD during trauma triggers increased activity and connectivity of the dlPFC and vlPFC involved in emotion regulation and executive functioning, findings supported by other rt‐fMRI‐NFB groups (Misaki et al., 2018b; Zotev et al., 2018). With regard to ICNs, Nicholson et al. (2018) also found that downregulating the amygdala with rt‐fMRI‐NFB led to increased recruitment of the CEN and stabilized DMN recruitment over NFB training. This represents a critical finding as individuals with PTSD have been shown to maladaptively recruit the DMN instead of the CEN during tasks that require cognitive control (Daniels et al., 2010). Of importance, Zotev et al. (2018) also showed in a randomized controlled study that amygdala regulation using rt‐fMRI‐NFB leads to significantly reduced PTSD severity scores, including significant reductions on avoidance, hyperarousal, and depressive symptoms. Extending the amygdala rt‐fMRI‐NFB literature, machine learning classifiers have also been utilized to improve performance on emotional conflict tasks by differentially selecting for brain states associated with targets as compared to trauma distractors (Weaver et al., 2020). Additionally, upregulating anterior cingulate cortex (ACC) activity has also been utilized in PTSD as a means to improve implicit emotion regulation capacities (Zweerings et al., 2018). Taken together, these results suggest that regulating specific brain areas tied to the manifestation of PTSD symptoms (e.g., the PCC of the DMN) may result in clinically meaningful changes, where additional studies are urgently needed to explore novel neurofeedback targets in PTSD.

### 1.1 Current study

Here, we utilized rt‐fMRI‐NFB to train PCC downregulation during emotion induction paradigms (presentation of trauma‐related/distressing words) among individuals with PTSD and healthy controls. The rationale of the current study to downregulate the PCC was threefold: (1) the PCC is highly associated with PTSD symptomatology which together with other DMN areas, displays hyperactivity when trauma memories become activated (Frewen et al., 2020; Thome et al., 2019); (2) regulating neural signals related to the PCC/DMN using EEG‐NFB has shown promising preliminary evidence in a randomized controlled trial (Nicholson et al., 2020b); and (3) the feasibility of downregulating amygdala activation using rt‐fMRI‐NFB in patients with PTSD has been demonstrated, which resulted in a shift toward normalization of DMN connectivity and reduced PTSD severity scores (Nicholson et al., 2016a, 2018; Zotev et al., 2018). Given the dynamic interplay between intrinsic brain networks (Menon, 2011), we hypothesized that PCC downregulation would lead to concomitant alterations in activation among regions within the DMN (e.g., mPFC), SN, and CEN (e.g., dlPFC involved in emotion regulation). We further predicted that NFB training would lead to decreased state PTSD/emotional symptoms. Moreover, given the well‐documented differences between PTSD and healthy controls with respect to DMN recruitment during both emotion induction paradigms and executive functioning tasks, we hypothesized unique neural mechanisms associated with regulation (i.e., psychopathological specificity) and predicted that machine learning models would be able to accurately classify PTSD versus healthy controls during NFB training.

## 2 METHODS

### 2.1 Participants

Our neuroimaging sample consisted of *n* = 30 individuals [*n* = 15 patients with a primary diagnosis of PTSD and *n* = 15 healthy participants (see Table 1 for demographic and clinical characteristics of the study sample)]. The sample size of this preliminary investigation was based on study feasibility during the time of recruitment. One participant in the PTSD group was excluded from the analyses since they reported having fallen asleep in the scanner during the transfer run, thus leaving the final sample size *n* = 14 in the PTSD group and *n* = 15 in the healthy control group. No individual had previously received NFB, and there was no sample overlap with our previous NFB investigations (Nicholson et al., 2016a, 2018). There were nonsignificant differences with respect to biological sex between the PTSD and healthy control groups. However, the mean age of participants in the PTSD group was significantly higher as compared to the healthy control group. Importantly, when age was included as a covariate within the analyses described below, our neural activation results were not significantly affected. Participants were recruited from 2017 to 2019 through referrals from family physicians, mental health professionals, psychology/psychiatric clinics, community programs for traumatic stress, and posters/advertisements within the London, Ontario community.

**TABLE 1:** Demographic and clinical information

	PTSD group	Healthy control group
***N***	14	15
***Sex***	6 females, 8 males	10 females, 5 males
***Years of age***	49.50 (± 5.11)	37.73 (±12.86)
***CAPS‐total***	43.21 (±8.26)	0 (±0)
***BDI‐total***	32.14 (±12.55)	1.2 (±2.46)
***CTQ‐total***	61.50 (±25.84)	31.13 (±8.44)
***MDI‐total***	87.36 (±28.23)	43.2 (±4.36)
***DERS‐total***	107.64 (±24.84)	52.80 (±9.03)
***MDD***	*Current = 9, past = 2*	*Current = 0, past = 0*
***Agoraphobia***	*Current = 1, past = 0*	*Current = 0, past = 0*
***Panic disorder***	*Current = 1, past = 0*	*Current = 0, past = 0*
***Somatization disorder***	*Current = 3, past = 0*	*Current = 0, past = 0*
***Psychotropic medication***	10	0

*Note*: Values in bracket indicate standard deviation.

Abbreviations: PTSD = Posttraumatic Stress Disorder, CAPS = Clinician Administered PTSD Scale, BDI = Becks Depression Inventory, CTQ = Childhood Trauma Questionnaire (*none or minimal childhood trauma = 25–36, moderate = 56–68, extreme trauma > 72*), MDI = Multiscale Dissociation Inventory, DERS = Difficulty in Emotion Regulation Scale, MDD = Major Depressive Disorder.

The inclusion criteria for PTSD participants included a primary diagnosis of PTSD as determined using the Clinician‐Administered PTSD Scale (CAPS‐5) and the Structured Clinical Interview for DSM‐5 (SCID) (First et al., 2002; Weathers et al., 2013). Patients with PTSD currently receiving psychotropic medication were on a stable dose for 1 month prior to their participation in the NFB study. Exclusion criteria for PTSD patients included alcohol or substance use disorder not in sustained full remission within the last 3 months prior to scanning and a lifetime diagnosis of bipolar or psychotic disorders. PTSD patients were also excluded from the study if they had prominent current suicidal ideation within the past 3 months or self‐injurious behaviours in the last 3 months requiring medical attention. Exclusion criteria for the healthy control group included lifetime psychiatric illness and current use of any psychotropic medications. Exclusion criteria for all participants included past or current biofeedback treatment, noncompliance with 3 Tesla fMRI safety standards, significant untreated medical illness, pregnancy, a history of neurological or pervasive developmental disorders, and previous head injury with loss of consciousness. Please see the supplementary materials section (Table S1) for a detailed report on the history of trauma exposure in each group.

Participants completed a battery of assessments before the NFB experiment, which consisted of the Beck's Depression Inventory (BDI) (Beck et al., 1997), the Childhood Trauma Questionnaire (CTQ) (Bernstein et al., 2003), and the Multiscale Dissociation Inventory (MDI) (Briere, 2002). In addition, in order to assess state changes in emotion‐related symptoms during NFB, participants completed the Response to Script Driven Imagery (RSDI) Scale (Hopper et al., 2007a) after each of the 4 fMRI runs, which consisted of the following symptom subscales: reliving, distress, physical reactions, dissociation, and numbing. All scanning took place at the Lawson Health Research Institute in London, Ontario, Canada. The study was approved by the Research Ethics Board at Western University, Canada, where participants gave written and informed consent and received financial compensation for participating in the study.

### 2.2 Neurofeedback paradigm

**FIGURE 1:**
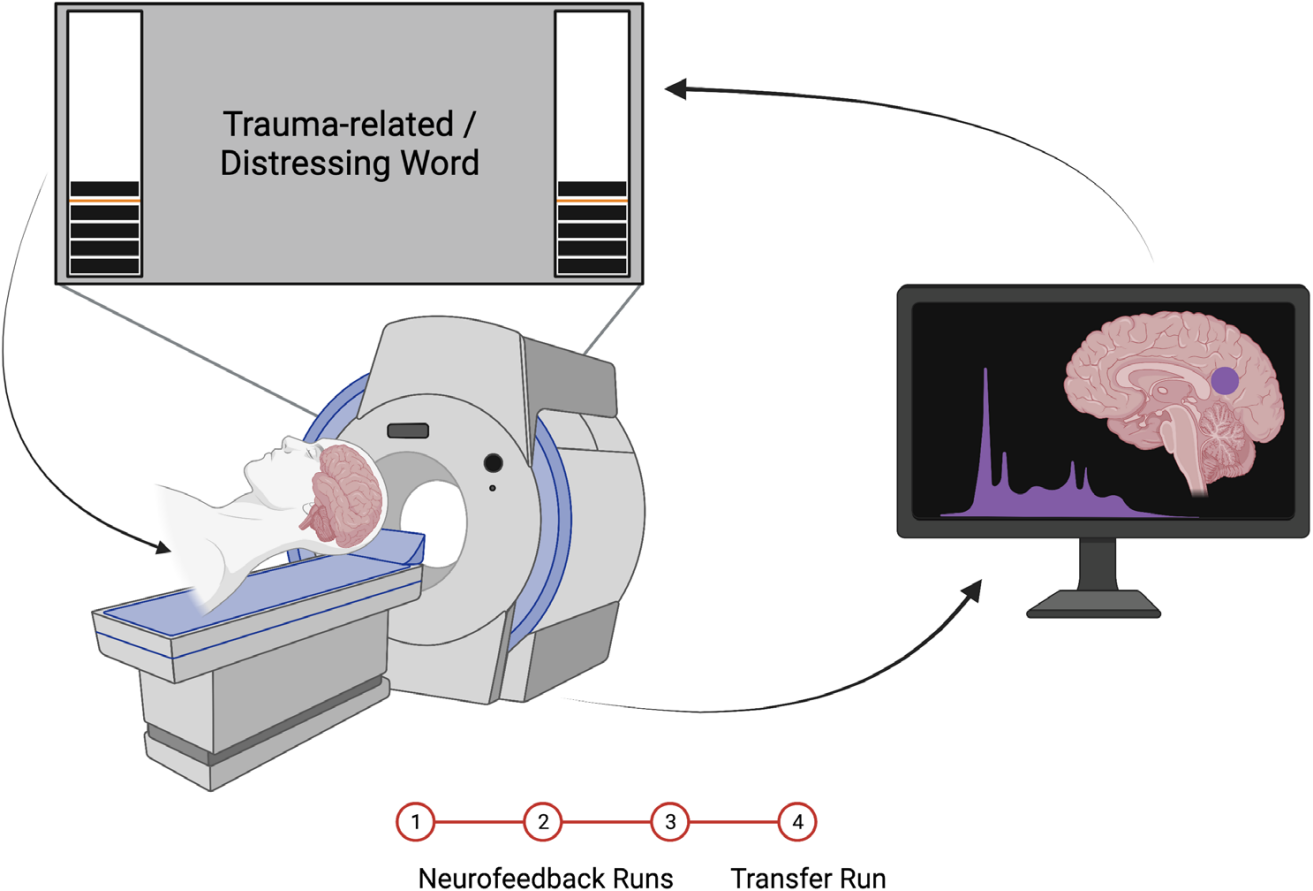
Schematic of the real‐time fMRI neurofeedback set‐up. Brain activity in the neurofeedback target region (posterior cingulate cortex) was processed in real‐time and presented to participants in the fMRI scanner as thermometers that increased or decreased as activation fluctuated. Participants completed three neurofeedback training runs and a transfer run without neurofeedback signal. Figure created with BioRender.com.

We implemented an experimental protocol and paradigm that was identical to our previous NFB investigations (Nicholson et al., 2016a, 2018); however, we trained individuals to downregulate the PCC as opposed to the amygdala (Figure 1). Participants were instructed that they would be *“regulating an area of the brain related to emotional experience*,” that is, to decrease activation within the PCC. In order to elicit unbiased and personalized regulatory strategies, specific instruction on how to regulate the brain region‐of‐interest was not provided (Paret et al., 2014, 2016a; Nicholson et al., 2016a, 2018; Zaehringer et al., 2019). During training trials, feedback of PCC activation was displayed to participants in the form of two identical thermometers on the left and right side of a screen projected inside the scanner. The bars on the thermometer increased/decreased as BOLD signal in the PCC target fluctuated, where an orange line on the thermometer indicated baseline PCC activation (Figure 1).

**FIGURE 2:**
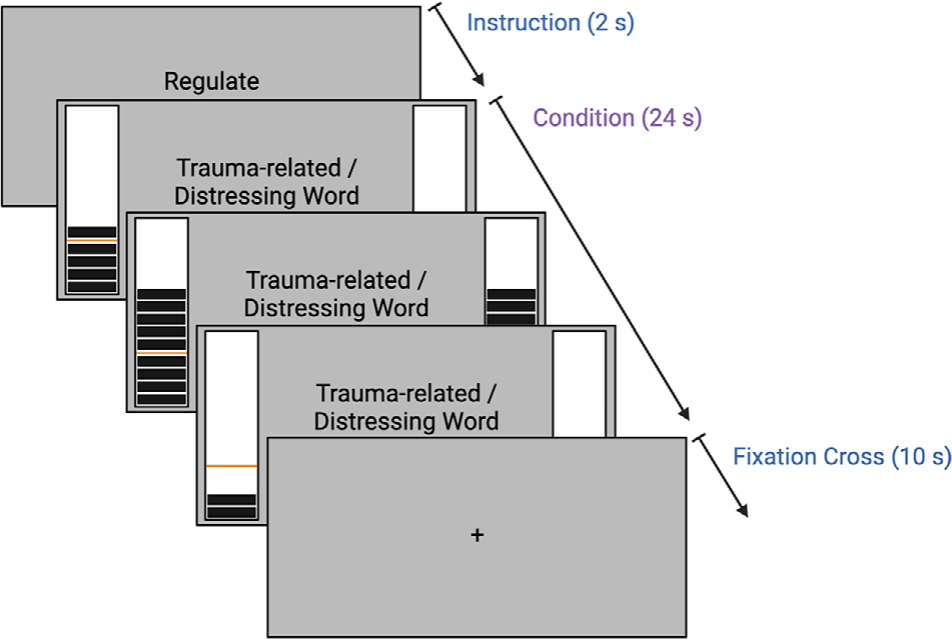
Neurofeedback experimental procedure for the regulate condition. The same timing was utilized for (i) view conditions in which participants viewed trauma‐related/distressing words while not attempting to regulate and (ii) for neutral conditions in which participants viewed neutral words and did not attempt to regulate. A trial started with a 2 s instruction slide indicating trial type (i.e., regulate, view, neutral). In the following block, participants saw either a trauma‐related/distressing word or a neutral word with a thermometer at both sides. The thermometer displayed the change in brain activation and was updated every 2 s.

Our neurofeedback protocol consisted of three conditions: (i) *regulate*, (ii) *view*, and (iii) *neutral*. During the *regulate* condition (Figure 2), individuals were asked to decrease activity in the brain target (decrease the bars on the thermometer corresponding to PCC activation) while viewing either personalized trauma‐related words for the PTSD group or a matched stressful word for the healthy control group (Nicholson et al., 2016a, 2018). During the *view* condition, individuals were asked to respond naturally to their personalized trauma/stressful words while not attempting to regulate the target brain area. *Neutral* trials consisted of asking individuals to respond naturally to personalized neutral words for both groups. Personalized trauma/stressful words (*n* = 10) and neutral words (*n* = 10) were selected by participants with a trauma‐informed clinician and matched on subjective units of distress to control for between subject/group variability. The personalized trauma words selected by participants with PTSD were related to individual experiences of trauma. Furthermore, personalized stressful words selected by healthy controls were related to the individual's most stressful life event. Stimuli were presented with Presentation software (Neurobehavioral Systems, Berkeley, CA). Participants were first provided with written instructions, followed by a single example trial within the scanner. Our experimental design then consisted of three consecutive neurofeedback training runs, which was followed by one transfer run in which individuals were presented with the same three conditions but without neurofeedback from the thermometer. Instructions were presented for 2 s before each condition; individual conditions lasted for 24 s and were followed by a 10 s implicit resting state where participants viewed a fixation cross (Figure 2). An experimental run lasted about 9 min and consisted of 15 trials (5 of each condition, counterbalanced and separated by an intertrial fixation cross) (Nicholson et al., 2016a, 2018).

One bar on the thermometer display corresponded to 0.2% signal change in the PCC, consisting of an upper activation range with a maximum of 2.8% signal change and a lower activation range with a maximum of 1.2% signal change (Paret et al., 2014, 2016b; Nicholson et al., 2018). Participants were instructed to visually focus on the word during its entire presentation and to view the two thermometers in their peripheral vision. Emotion‐induction effects of personalized stimuli were confirmed both on the subjective experience level via inspection of RSDI scores and on the neurobiological level by contrasting *view* as compared to *neutral* conditions (see results section below). Participants were also informed of the temporal delay that would occur during neurofeedback, corresponding to both the BOLD signal delay and real‐time processing of this neural activation. Finally, when a neurofeedback run was completed, individuals were asked to rate their perceived ability to regulate the target brain area. Specifically, we asked participants to rate the extent to which they were able to gain control over the neurofeedback signal, which ranged from 0 (not at all) to 6 (a great deal).

### 2.3 Real‐time signal processing for neurofeedback

Anatomical scans were first imported into BrainVoyager (Brain Innovations, Maastrict, the Netherlands), skull‐stripped, and then transformed into Talairach space. Normalization parameters were then loaded into TurboBrainVoyager (TBV) (Brain Innovations, Maastricht, the Netherlands). Motion correction features and spatial smoothing using a 4‐mm full‐width‐half‐maximum (FWHM) Gaussian kernel were implemented in TBV, and the initial 2 volumes of the functional scans were discarded before real‐time processing. We defined the target PCC using a 6 mm sphere over the following coordinate (MNI: 0 ‐50 20) (Bluhm et al., 2009). We used the “best voxel selection” tool in TBV to calculate the BOLD signal amplitude in the PCC. This tool identifies the 33% most active voxels for the *view* > *neutral* contrast. Further details on dynamic ROI definitions can be found in our previous publications (Nicholson et al., 2016a, 2018). The first two trials of each neurofeedback run consisted of *view* and *neutral* conditions thereby allowing for initial selection of PCC voxels based on the *view* > *neutral* contrast, which was dynamically updated as voxels selection was refined along the course of training. For each trial, the mean of the last 4 data points before stimuli onset (during the implicit resting state) were selected as a baseline and indicated to participants as an orange line on the thermometer display. The signal was smoothed by calculating the mean of the current and the preceding 3 data points (Paret et al., 2014, 2016b; Nicholson et al., 2016a).

### 2.4 fMRI image acquisition and preprocessing

Neuroimaging was conducted using a 3 Tesla MRI Scanner at the Lawson Health Research Institute (Siemens Biograph mMR, Siemens Medical Solutions, Erlangen, Germany) with a 32‐channel head coil, where during scanning participants’ heads were stabilized. Functional whole brain images of the BOLD contrast were acquired with a gradient echo T2*‐weighted echo‐planar‐imaging sequence (TE = 30 ms, TR = 2 s, FOV = 192 × 192 mm, flip angle = 80°, inplane resolution = 3 × 3 mm). One volume comprised 36 ascending interleaved slices tilted −20° from AC‐PC orientation with a thickness of 3 mm and slice gap of 1 mm. The experimental runs comprised 284 volumes each, where T1‐weighted anatomical images were acquired with a Magnetization Prepared Rapid Acquisition Gradient Echo sequence (TE = 3.03 ms, TR = 2.3 s, 192 slices and FOV = 256 × 256 mm).

Preprocessing of the functional images was performed with SPM12 (Wellcome Department of Cognitive Neurology, London, UK) within MATLAB R2020a. Our standard preprocessing routine included discarding 4 initial volumes, slice time correction to the middle slice, reorientation to the AC‐PC axis, spatial alignment to the mean image using a rigid body transformation, reslicing, and coregistration of the functional mean image to the subject's anatomical image. The coregistered images were segmented using the “New Segment” method implemented in SPM12. The functional images were normalized to MNI space (Montréal Neurological Institute) and were smoothed with a FWHM Gaussian kernel of 6 mm. Additional correction for motion was implemented using the ART software package (Gabrieli Lab, McGovern Institute for Brain Research, Cambridge, MA), which computes regressors that account for outlier volumes.

### 2.5 Statistical analyses

#### 2.5.1 First‐level analysis

We defined separate sessions for each neurofeedback training run and the transfer run, where all events (initial rest, instructions, fixation, and conditions) were modeled as blocks of brain activation and convolved with the hemodynamic response function. In the first level, functional data were also high‐pass filtered and serial correlations were accounted for using an autoregressive model. Additionally, ART software regressors were included as nuisance variables to account for any additional movement and outlier artifacts. The three experimental conditions (*regulate, view, and neutral*) were modeled separately on the first level.

#### 2.5.2 Second‐level analyses

We first conducted a split‐plot full factorial 2 (group) by 3 (condition) by 3 (NFB training run) ANOVA within SPM12 to investigate changes in whole‐brain activation, inputting separate condition specific contrast images generated in the first level. As we were specifically interested in differential activation during the *regulate* and *view* conditions (Nicholson et al., 2016a; 2018), we examined follow‐up comparisons focusing on between condition effects within group, as well as between groups comparing the PTSD and healthy control groups. We then examined the transfer run separately, where we conducted a 2 (group) by 3 (condition) ANOVA and subsequently examined aforementioned direct follow‐up comparisons. All analyses were whole‐brain corrected for multiple comparisons using a clusterwise false discovery rate (FDR) threshold at *p* < .05, *k* = 10, with an initial clustering defining threshold in SPM at *p* < .001, *k* = 10 (Eklund et al., 2016; Roiser et al., 2016).

Finally, we conducted linear regression analyses across all subjects, examining potential correlations between trait‐based symptoms and whole‐brain activation during *view* as compared to *regulate* conditions over NFB training runs. Here, we examined PTSD symptom severity scores (CAPS‐5 total), difficulty in emotion regulation scores (DERS total), and depressive symptoms (BDI total).

#### 2.5.3 Neurofeedback PCC downregulation analysis

**FIGURE 3:**
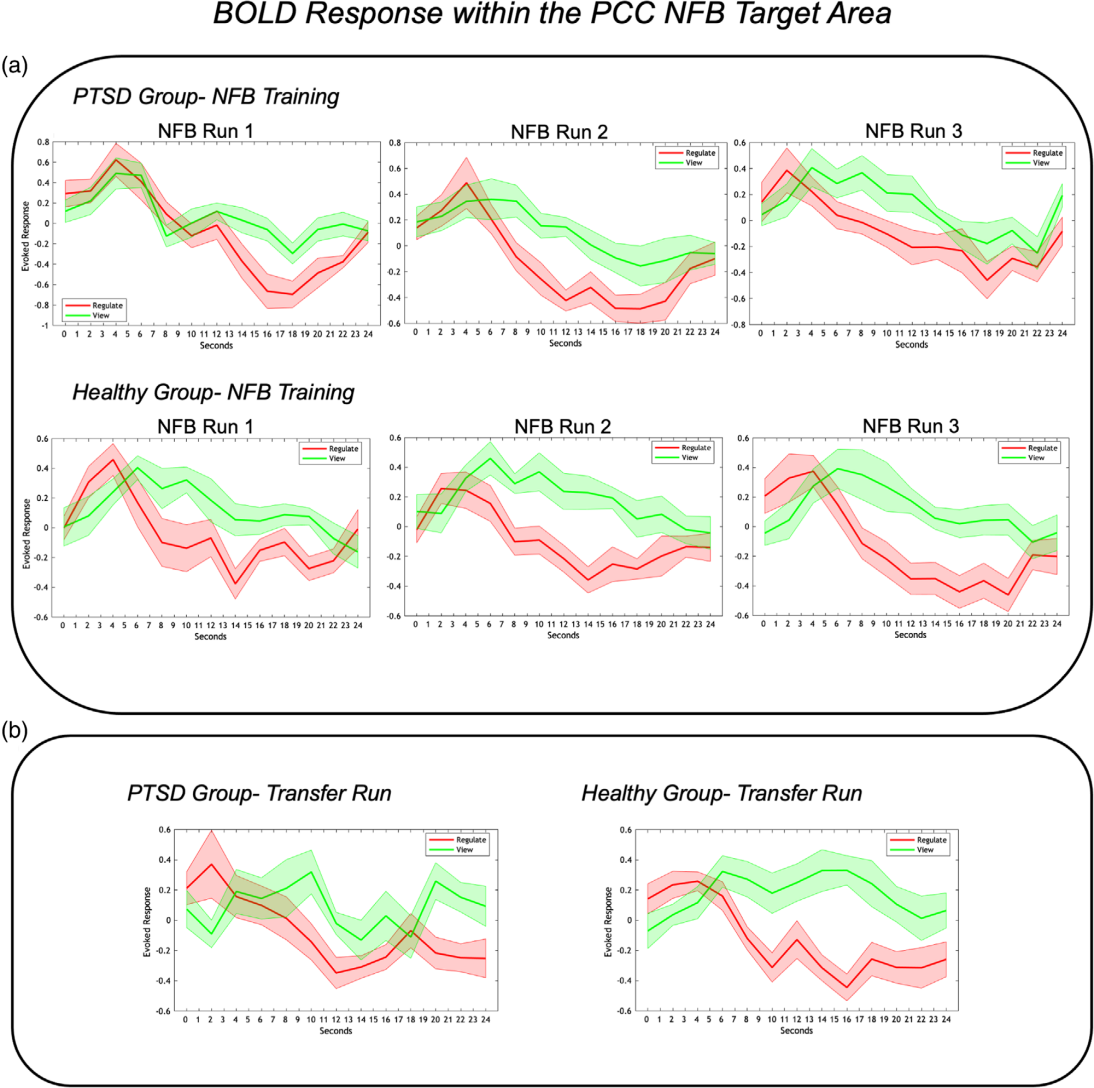
(a) Event‐related BOLD response in the NFB target area (PCC), during the three training runs in the PTSD and healthy control groups. The red lines indicate PCC activation during the *regulate* condition, where the goal was to decrease activation while viewing trauma/stressful words. The green lines indicate PCC activation during the *view* condition, where participants were not attempting to decrease activation while viewing trauma/stressful words. Here, PCC activation was significantly lower during *regulate* as compared to *view* conditions for NFB training runs 1–3, for both the PTSD and healthy control groups. (b) Event‐related BOLD response in the target area (PCC) during the transfer run when neurofeedback was not provided. Taken together, this demonstrates that both groups were able to gain control over downregulating their PCC with similar success. The *x*‐axis of the graphs indicate time over the 24 s conditions; the *y*‐axis indicates the event‐related BOLD response (peristimulus time histogram) in the target area. Shaded areas of red and green indicate standard error of the mean. Abbreviations: PCC = posterior cingulate cortex, NFB = neurofeedback.

In order to evaluate PCC downregulation (neurofeedback success), we extracted the event‐related BOLD response (peristimulus time histogram) from the PCC target area during the *regulate* and the *view* conditions using rfxplot software (Gläscher, 2009), using the same sphere definition that was used to generate feedback for participants in the fMRI scanner. Here, we extracted the event‐related BOLD response from individual peaks within the search volume, and these values were then passed to SPSS (v.26) for statistical analyses. Within rfxplot software, event‐related BOLD responses display the average height of the BOLD responses within a defined volume and time window (Gläscher, 2009). Rfxplot shows the actual data and does not rely on first‐ or second‐level models. Event‐related BOLD responses are estimated by a condition‐specific Finite Impulse Response (FIR) model (Gläscher, 2009). Here, the condition duration in which the BOLD response is expected to fluctuate is parcellated into temporal bins (TR = 2 s) starting at the onset of all trials belonging to a particular condition. The parameter estimate for each bin of the FIR model is identical to the mean BOLD response in that bin, thus creating an event‐related BOLD time course for each subject. For the final display (Figure 3), rfxplot averages subject‐specific event‐related BOLD responses based on group.

For the PTSD and healthy control groups separately, we computed repeated measures 2 (condition) by 4 (NFB run) ANOVAs. Subsequently, we then conducted a priori defined paired sample *t*‐tests, comparing the average BOLD response within the NFB target area between conditions for each NFB run within groups. We also conducted independent samples *t*‐tests comparing the average BOLD response within the NFB target area during a single NFB run for a given condition between groups. Lastly, we conducted repeated measures one‐way ANOVAs for the regulate condition for each group in order to examine potential learning effects across NFB training.

#### 2.5.4 State changes in emotional experience over neurofeedback

We examined state changes in subjective response to traumatic/stressful stimuli over the NFB training experiment, as measured by RSDI subscales. As collected data were not normally distributed, we computed nonparametric Friedman's repeated measures ANOVAs for each group for each RSDI subscale. Here, we Bonferroni corrected our statistical threshold (*p* < .05/5 = .01) for nonparametric ANOVAs. Paired comparisons between time points were conducted using nonparametric tests for related samples (Wilcoxon signed‐ranks test). We then compared state symptoms across NFB runs between groups with Mann–Whitney U tests.

#### 2.5.5 Machine learning classification analysis

We examined the accuracy of machine learning algorithms in classifying PTSD patients as compared to healthy individuals based on whole brain activation during *view* as compared to *regulate* conditions across NFB training runs. Here, we implemented L1‐Multiple Kernel Learning (MKL) Classification algorithms within PRoNTo toolbox (http://www.mlnl.cs.ucl.ac.uk/pronto/) (Schrouff et al., 2013) running under Matlab2020a (Mathworks, 2020). This approach has two potential benefits: (1) it can lead to improved overall generalization performance and (2) it can identify a sparse subset of relevant brain regions for the predictive model. Predictors consisted of contrast images corresponding to the *view* as compared to the *regulate* condition, where each NFB training run was inputted as a separate modality during design specification. A feature set was then prepared; NFB runs were concatenated and separate kernels were built for each modality and for each anatomical region defined by the Automated Anatomical Labeling (AAL, Tzourio‐ Mazoyer et al., 2002) atlas. Here, an L1‐MKL Classifier (Schrouff et al., 2018) was used to test if neural activation during *view* as compared to *regulate* could accurately predict group membership classification of PTSD versus healthy control. Critically, PRoNTo software implements kernel methods as a result of the high dimensionality of pattern vectors in neuroimaging data relative to the number of subjects (for more information see Schrouff et al., 2013). We used a leave‐one‐subject‐out (LOSO) cross‐validation procedure to estimate the generalizability of our classifiers, where features were mean‐centered and normalized. Statistical significance of classification accuracy measures was determined via permutation testing (20,000 permutations). We then used anatomical/functional information from the AAL atlas to learn the contribution of each brain region to decision function of the machine, a function afforded by the grouping structure and the implementation of a spare version of Multiple Kernel Learning within PRoNTo toolbox (Schrouff et al. 2018).

## 3 RESULTS

### 3.1 PCC downregulation with neurofeedback

We found that both patients with PTSD and healthy individuals were able to significantly downregulate their PCC during *regulate* as compared to *view* conditions (Figure 3).

In summary, the average event‐related BOLD response within the PCC (NFB target area) was significantly lower during the *regulate* as compared to *view* conditions for all three NFB training runs (see Figure 3a), as well as for the transfer run (see Figure 3b) in both the PTSD and healthy control group, where activation during *regulate* did not differ significantly when comparing NFB runs directly.

Specifically, our 2 (condition) by 4 (NFB run) repeated measures ANOVAs yielded significant main effects of condition for analyses conducted within the PTSD group (*F*(1, 13) = 37.6, *p* < .0001, η^2^ = .743) and within the control group (*F*(1, 14) = 33.67, *p* < .0001, η^2^ = .706). We found nonsignificant main effects of NFB run and nonsignificant interactions for both groups. Next, follow‐up paired sample *t*‐tests demonstrated that the average event‐related BOLD response during the *regulate* condition was significantly lower than the *view* condition for all NFB training runs and the transfer run, for both the PTSD group (NFB run 1: *t*(13) = −3.50, *p* = .004; NFB run 2: *t*(13) = −3.00, *p* = .011; NFB run 3: *t*(13) = −3.71, *p* = .003; transfer run: *t*(13) = −3.50, *p* = .004) and the healthy control group (NFB run 1: *t*(14) = −3.63, *p* = .003; NFB run 2: *t*(14) = −5.43, *p* < .0001; NFB run 3: *t*(14) = −5.77, *p* < .0001; transfer run: *t*(14) = −3.21, *p* = .006). Interestingly, when comparing each condition during a respective NFB run and during the transfer run between groups, we found nonsignificant differences in the average event‐related BOLD response during the *regulate* and *view* conditions between PTSD and healthy control groups. When conducting the same analysis but investigating the difference in BOLD response between the *regulate* and *view* conditions between PTSD and healthy control groups, we also found nonsignificant differences between groups. Indeed, this implies that both experimental groups were able to gain control over downregulating their PCC with similar success. However, as demonstrated below, the neural mechanisms by which the regulation was achieved is starkly different. Finally, when examining repeated measures one‐way ANOVAS for *regulate* trials over the NFB runs, we report nonsignificant main effects of run for both the PTSD and healthy control groups. Please see the supplementary materials section (Figure S1) for plots of the event‐related BOLD response within the PCC NFB target region during all conditions (*neutral*, *view*, and *regulate*).

When evaluating perceived ability to regulate the neurofeedback signal, our 2 (group) by 4 (run) split‐plot repeated measured ANOVA revealed a nonsignificant group by run interaction, where the PTSD and healthy control groups did not differ significantly when comparing each run directly (PTSD: *M* = 2.5, *SD* ±1.42; healthy control group: *M* = 2.83, *SD* ±1.21; perceived ability to regulate scale 0 = not at all, 6 = a great deal). Additionally, within‐group comparisons of subjective ratings on ability to regulate the neurofeedback signal at run 1 versus run 4 were found to be nonsignificant.

### 3.2 Differential mechanisms of PCC downregulation: Neural activation analysis

Our split‐plot ANOVA revealed a significant group by condition by NFB run interaction within the right dlPFC (see Table 2). This fortified the examination of our subsequent a priori planned comparisons. Here, we focused on investigation around the difference between the *regulate* and *view* conditions. Importantly, however, when examining neural activation during *view* as compared to *neutral* conditions across all participants, we found significant activation within the PCC/precuneus (NFB target area), the left anterior insula, the bilateral cerebellum (lobule VI and Crus I/II), the left dmPFC, and the left angular gyrus (see supplemental material Table S2). These findings confirm increased neural activation within the NFB target region (PCC) as a result of the emotion induction paradigm and provide construct validity to the current investigation in both the PTSD and healthy control groups.

**TABLE 2:** 2 (Group) × 3 (condition) × 3 (NFB Run) split plot ANOVA

Comparison	Brain region	H	Cluster size	MNI coordinate			*F* stat.	Z score	*p‐FDR* cluster level
				*x*	*y*	*z*			
Group × condition × run interaction	Dorsolateral PFC	R	138	44	38	30	8.33	4.56	.011

*Note*: Results of the full factorial split plot 2 (group) by 3 (condition) by 3 (NFB run) ANOVA evaluated at the FDR‐cluster corrected threshold for multiple comparisons (*p* < .05, *k* = 10).

**FIGURE 4:**
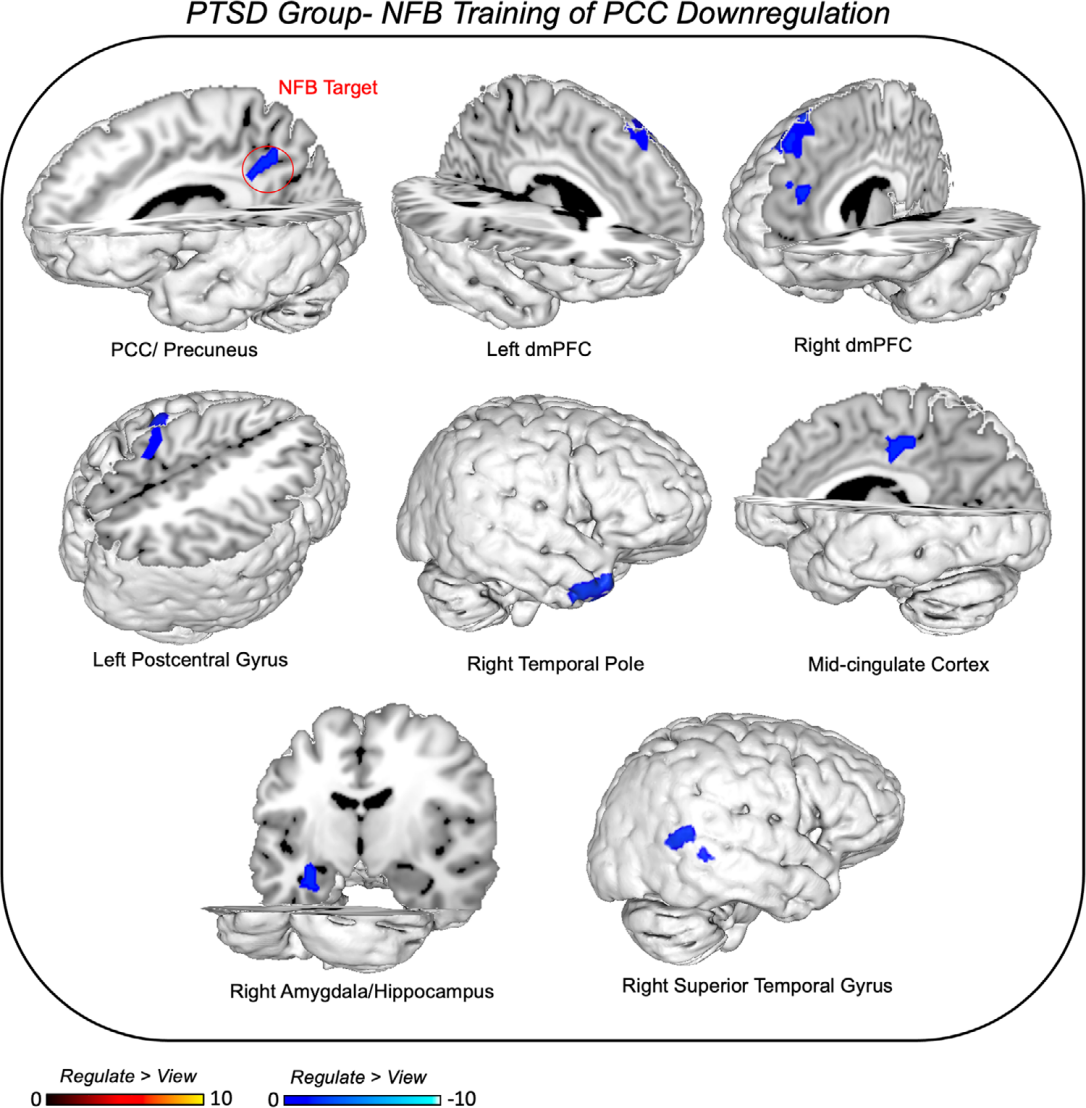
Neural dynamics during NFB training of PCC downregulation. Illustration of brain areas that show concomitant decreases in activity during *regulate* as compared to *view* conditions across NFB training runs in the PTSD group. Results evaluated at the FDR‐cluster corrected level for multiple comparisons (*p* < .05, *k* = 10). Abbreviations: PCC = posterior cingulate cortex, dmPFC = dorsomedial prefrontal cortex.

**FIGURE 5:**
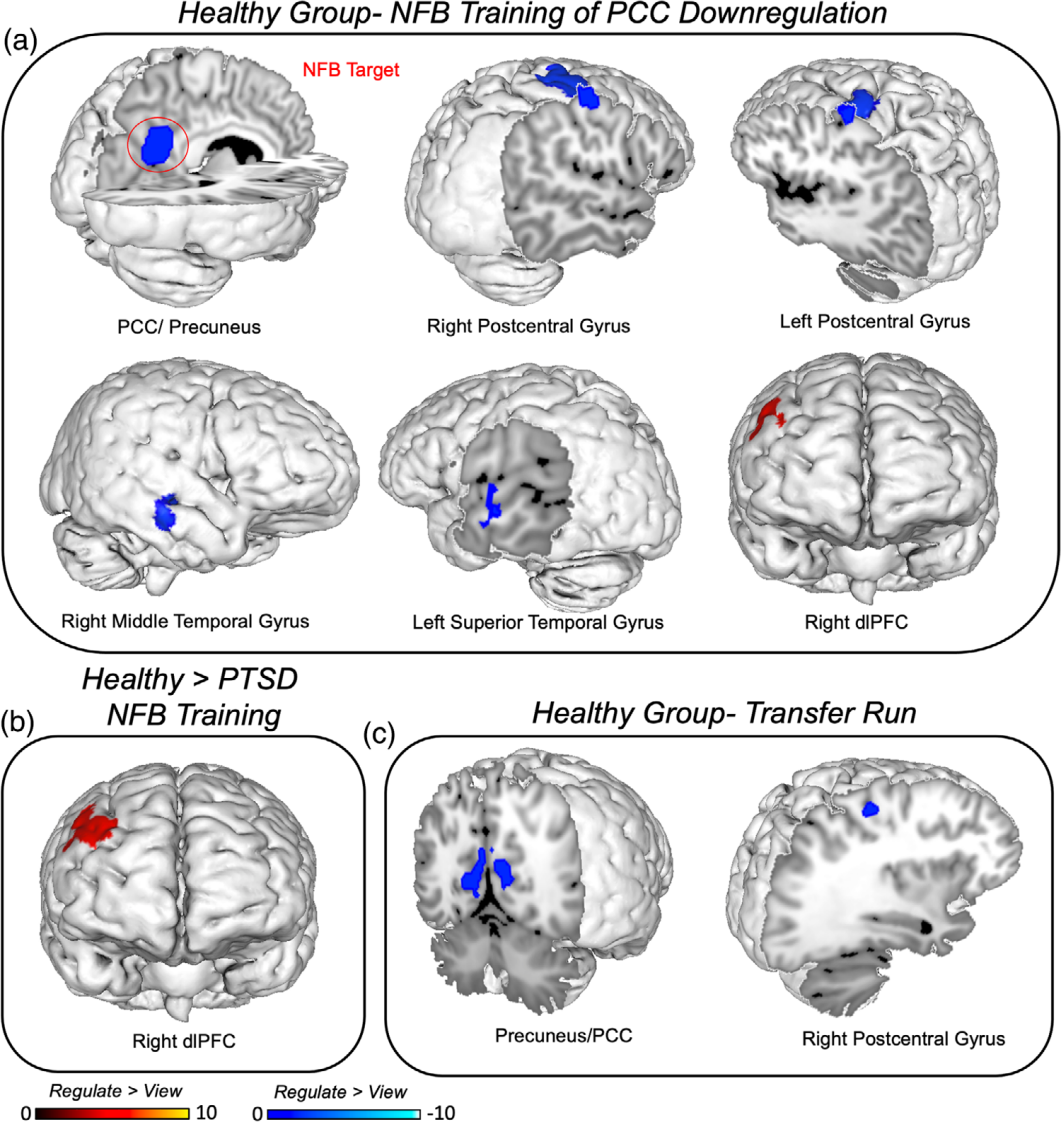
(a) Neural dynamics during NFB training of PCC downregulation. Brain areas that show concomitant decreases (blue) and increases (red) in activity during *regulate* as compared to *view* conditions across NFB training runs in the healthy control group. (b) Direct group comparisons revealed that the healthy control group displayed increased right dlPFC activation relative to the PTSD group, during *regulate* as compared to *view* conditions over NFB training. (c) Illustration of brain areas that show concomitant decreases (blue) in activity during *regulate* as compared to *view* conditions during the transfer run without neurofeedback in the healthy control group. Results evaluated at the FDR‐cluster corrected level for multiple comparisons (*p* < .05, *k* = 10). Abbreviations: NFB = neurofeedback, PCC = posterior cingulate cortex, dlPFC = dorsolateral prefrontal cortex.

When examining NFB training runs within groups, during *regulate* as compared to *view* conditions, the PTSD group showed expected decreases in the PCC/precuneus target area, as well as concomitant decreases in the bilateral dmPFC, the left postcentral gyrus, the right temporal pole, the mid‐cingulate cortex, the left amygdala/hippocampus, and the right superior temporal gyrus (see Table 3 and Figure 4). When analyzing NFB training runs during *regulate* as compared to *view* conditions, the healthy control group also showed expected decreases in the PCC/precuneus target area, in addition to concomitant decreases in the bilateral postcentral gyrus, the right middle temporal gyrus, and the left superior temporal gyrus (see Table 3 and Figure 5a). Additionally, the healthy control group exhibited increased activation during *regulate* as compared to *view* conditions within the right dlPFC. Interestingly, direct group comparisons revealed that the healthy control group displayed increased right dlPFC activation relative to the PTSD group during *regulate* conditions as compared to *view* conditions (see Table 3 and Figure 5b).

**TABLE 3:** Neurofeedback training direct comparisons

Comparison	Contrast	Brain region	H	Cluster size	MNI coordinate			*t* Stat.	Z score	*p‐FDR* cluster level
					*x*	*y*	*z*			
**Within‐group**										
PTSD	Reg < View	Postcentral gyrus	L	149	–12	–33	76	5.16	4.95	=.011
		dmPFC	L	394	–16	32	58	4.72	4.56	<.001
		dmPFC	R	380	12	46	34	4.39	4.26	<.001
		Temporal pole	R	174	52	10	–30	4.49	4.38	=.006
		Mid‐cingulate cortex		265	4	–14	46	4.27	4.15	=.001
		Posterior cingulate cortex/precuneus		370	–2	–45	32	4.24	4.12	<.001
		Amygdala/hippocampus	L	100	–34	–16	–20	4.08	3.97	<.05
		Superior temporal gyrus	R	106	64	–42	10	3.86	3.77	<.05
	Reg > View	*ns*								
Healthy controls	Reg < View	Postcentral gyrus	R	684	40	–14	58	5.16	4.96	<.001
		Posterior cingulate cortex/precuneus		874	0	–62	22	4.90	4.72	<.001
		Postcentral gyrus	L	187	–36	–26	66	4.75	4.59	=.003
		Middle temporal gyrus	R	105	60	–8	–14	4.12	4.02	=.024
		Superior temporal gyrus	L	116	–62	–20	–2	3.97	3.87	=.020
	Reg > View	dlPFC	R	141	32	34	32	4.09	3.98	=.030
**Between‐group**										
Healthy controls > PTSD	Reg > View	dlPFC	R	175	32	52	30	4.26	4.04	=.019

*Note*: Results of the direct follow‐up comparisons during the NFB training runs, within and between the PTSD and healthy control groups, evaluated at the FDR‐cluster corrected threshold for multiple comparisons (*p* < .05, *k* = 10).

When examining neural activation during the transfer run within group, during *regulate* as compared to *view* conditions, the healthy control group displayed decreased precuneus/PCC and right postcentral gyrus activation (see Table 4 and Figure 5c). During the transfer run, we found nonsignificant differences between the *regulate* and *view* conditions within the PTSD group and when comparing these conditions in the transfer run between the PTSD and healthy control groups at a conservative FDR‐corrected threshold. Notably, when age was included as covariate, the aformentioned neural activation results were not significantly affected.

**TABLE 4:** Transfer run direct comparisons

Comparison	Contrast	Brain region	H	Cluster size	MNI coordinate			*t* Stat.	Z score	*p‐FDR* cluster level
					*x*	*y*	*z*			
**Within‐group**										
PTSD	Reg < View	*ns*								
	Reg > View	*ns*								
Healthy controls	Reg < View	Precuneus/PCC		870	–8	–60	12	4.67	4.26	<.001
		Postcentral gyrus	R	191	50	–18	54	4.21	3.90	=.006
	Reg > View	*ns*								
**Between‐group**										
Healthy controls > PTSD	Reg > View	*ns*								
	Reg < View	*ns*								

*Note*: Results of the direct follow‐up comparisons during the transfer runs, within and between the PTSD and healthy control groups, evaluated at the FDR‐cluster corrected threshold for multiple comparisons (*p* < .05, *k* = 10).

### 3.3 Clinical correlations with neural activation

**FIGURE 6:**
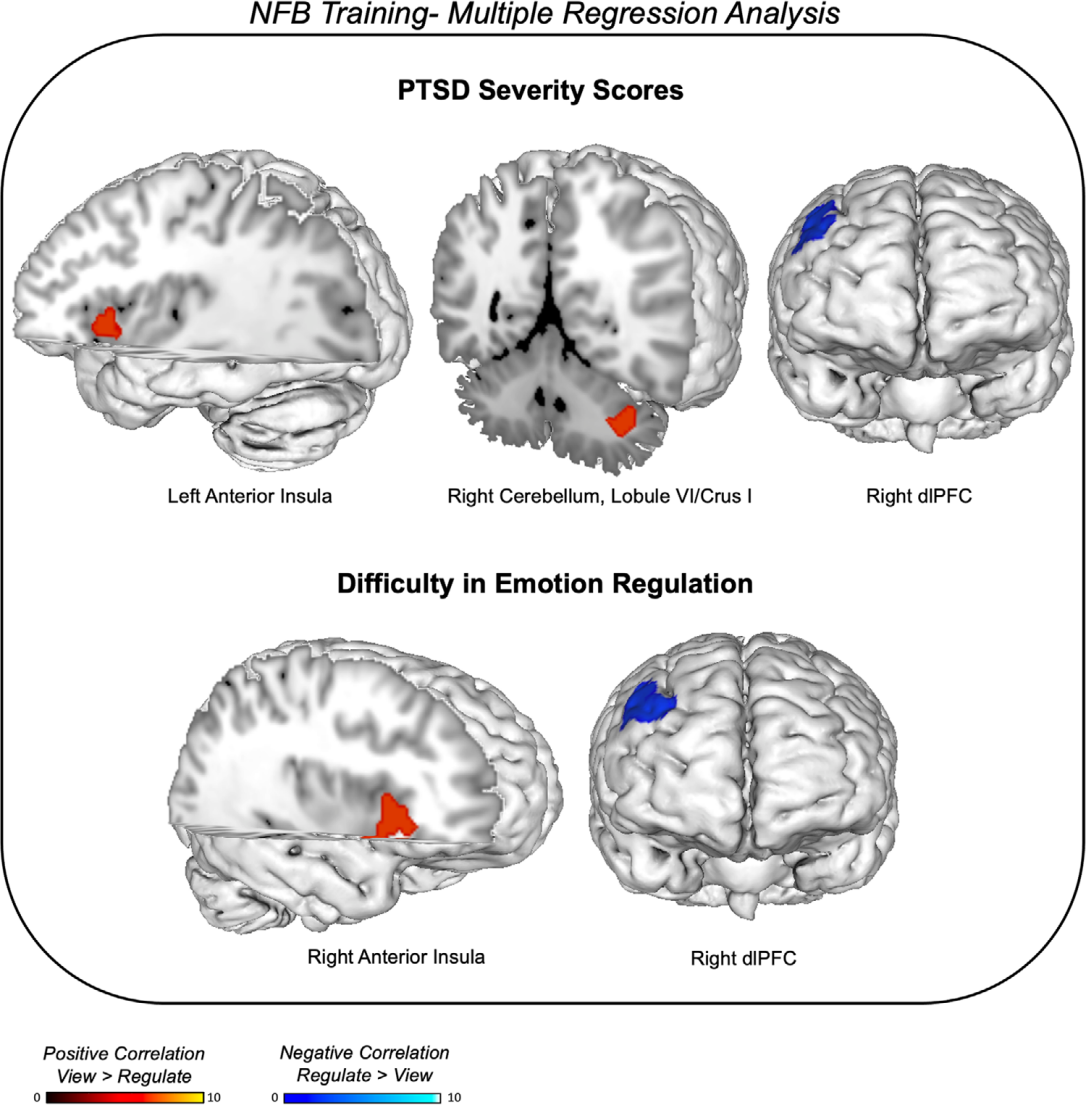
The upper portion of the figure shows correlations between PTSD severity scores (CAPS total) and brain activation during NFB training. The lower portion of the figure illustrates correlations between difficulties in emotion regulation (DERS total) and brain activation during NFB training. Red clusters indicate positive correlations during *view* as compared to *regulate* conditions. Blue clusters indicate negative correlations during *regulate* as compared to *view* conditions. During NFB training, the more severe the PTSD symptoms, the more the left anterior insula and the right cerebellum (lobule VI/Crus I) was activated during *view* as compared to *regulate* conditions. Additionally, the more the dlPFC was activated during *regulate* as compared to *view* conditions over NFB training, the less severe the PTSD symptoms. Furthermore, the more difficult it was for participants to regulate their emotions, the more the anterior insula was activated during *view* as compared to *regulate* conditions. Finally, the more dlPFC activation during *regulate* as compared to *view* conditions over NFB training, the less difficulties participants had with emotion regulation. Results evaluated at the FDR‐cluster corrected level for multiple comparisons (*p* < .05, *k* = 10). NFB = neurofeedback, dlPFC = dorsolateral prefrontal cortex.

When evaluating associations between neural activation and clinical measures with linear regression analyses, we found that PTSD severity scores (CAPS total) positively correlated with left anterior insula and right cerebellum (lobule VI/Crus I) activation during *view* as compared to *regulate* conditions (see Table 5 and Figure 6). In other words, the higher the PTSD symptoms, the more the left anterior insula and the right cerebellum (lobule VI/Crus I) was activated during the viewing of trauma/stressful words as compared to regulating the PCC while viewing these words. Additionally, PTSD severity scores (CAPS total) negatively correlated to activation in the right dlPFC during *regulate* as compared to *view* conditions. Hence, the more the dlPFC was activated during *regulate* as compared to *view* conditions, the less severe the PTSD symptoms. Considering significant correlations observed with CAPS‐total scores, we then conducted follow‐up post hoc linear regression analyses with scores related to the severity of intrusion symptoms (CAPS‐5 cluster B), severity of negative alterations in cognitions and mood (CAPS‐5 cluster D), and severity of arousal and reactivity symptoms (CAPS‐5 cluster E). Here, similar positive correlations were found with the bilateral anterior insula and the right cerebellum (lobule VI/Crus I) during *view* as compared to *regulate* conditions, as well as negative correlations with right dlPFC activation during *regulate* as compared to *view* conditions.

**TABLE 5:** Neurofeedback training multiple regression analysis

Measure	Contrast	Correlation	Brain region	H	Cluster size	MNI coordinate			*t* Stat.	Z score	*p‐FDR* cluster level
						*x*	*y*	*z*			
PTSD severity scores (CAPS total)	View > Reg	Positive	Anterior insula	L	123	–36	14	2	5.44	4.43	.040
			Cerebellum lobule VI/Crus I	R	169	36	–56	–28	4.90	4.11	.023
	Reg > View	Negative	dlPFC	R	152	38	44	32	5.11	4.24	.023
Intrusion symptoms (CAPS Cluster B)	View > Reg	Positive	Cerebellum lobule VI/Crus I	R	219	34	–50	–30	4.77	4.03	.022
	Reg > View	Negative	dlPFC	R	171	36	44	32	4.38	3.77	.037
Negative alterations in cognitions and mood (CAPS Cluster D)	View > Reg	Positive	Anterior Insula	L	139	–34	14	2	5.76	4.61	.032
			Cerebellum lobule VI/Crus I	R	160	36	–52	–28	5.17	4.27	.024
	Reg > View	Negative	dlPFC	R	192	36	44	32	5.33	4.37	.018
Arousal and reactivity symptoms (CAPS Cluster E)	View > Reg	Positive	Anterior Insula	L	144	–36	14	2	5.94	4.71	.020
			Anterior Insula	R	144	38	18	–2	5.19	4.28	.020
	Reg > View	Negative	dlPFC	R	175	38	44	32	5.54	4.49	.020
Difficulty in emotion regulation (DERS total)	View > Reg	Positive	Anterior insula	R	234	36	20	0	5.64	4.55	.004
	Reg > View	Negative	dlPFC	R	142	38	44	30	5.17	4.28	.029

*Note*: Results of the multiple regression analyses correlating clinical measures (CAPS total scores, CAPS symptom cluster scores, and DERS total) with neural activation during NFB training, evaluated at the FDR‐cluster corrected threshold for multiple comparisons (*p* < .05, *k* = 10). Follow‐up correlations with CAPS symptom cluster scores were conducted post hoc in light of significant associations with CAPS‐total scores.

Additionally, we found a positive correlation between difficulty in emotion regulation scores (DERS total) and the right anterior insula during *view* as compared to *regulate* conditions (see Table 5 and Figure 6). In other words, the more difficult it was for participants to regulate their emotions, the more the anterior insula was activated during the viewing of trauma/stressful words as compared to regulating the PCC while viewing these words. Furthermore, difficulty in emotion regulation was negatively correlated to right dlPFC activation during *regulate* as compared to *view* conditions. Indeed, the more dlPFC activation during *regulate*, the less difficulties participants had in emotion regulation.

### 3.4 State changes in emotional experience over neurofeedback

**FIGURE 7:**
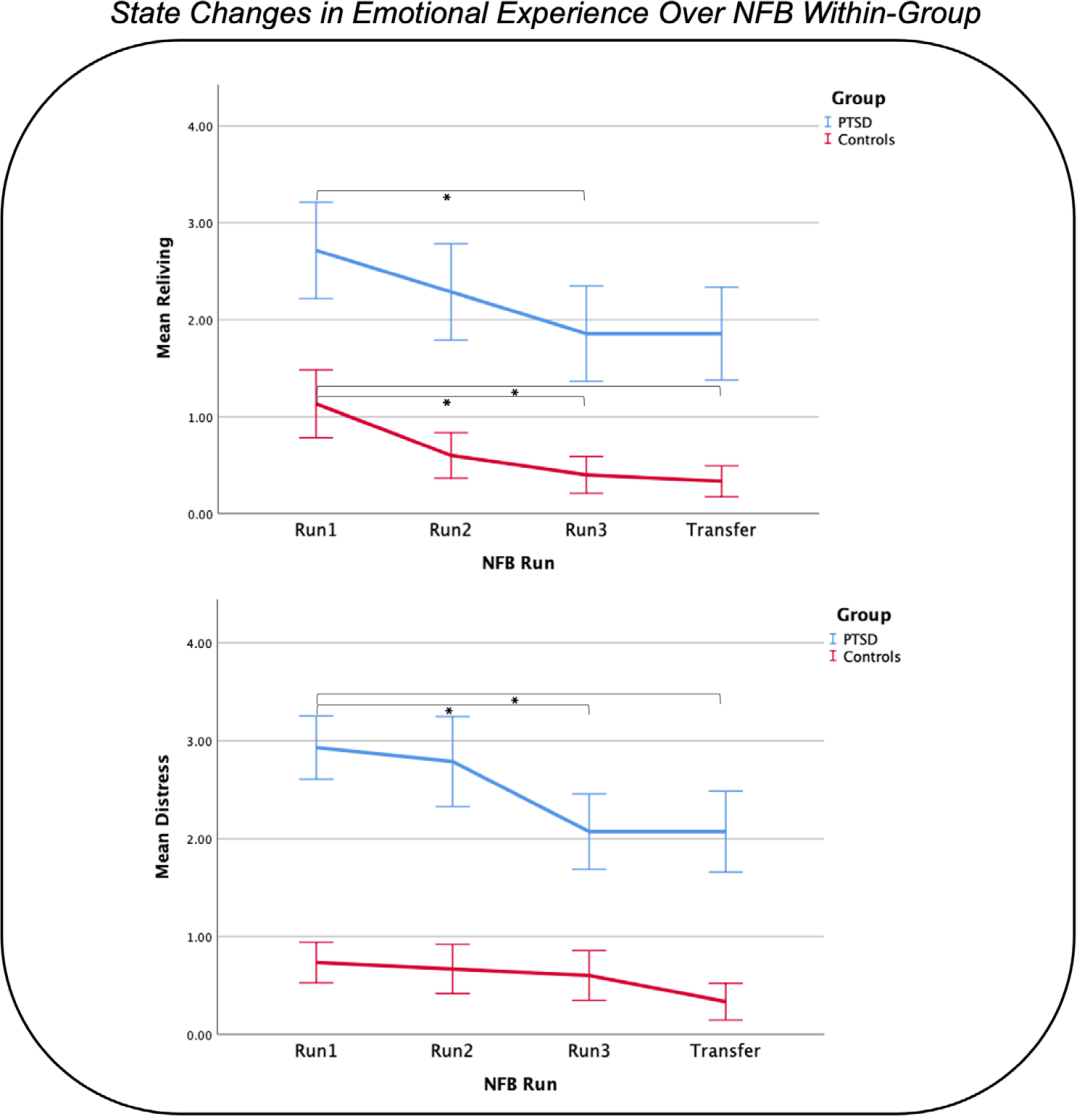
When examining state changes in emotional experience over NFB training in response to trauma/stressful stimuli presentation, we found that the PTSD and healthy control groups demonstrated significant reductions on reliving symptoms, where additionally, the PTSD group demonstrated significant reductions on distress symptoms as measured by the RSDI Scale. Abbreviations: NFB = neurofeedback, RSDI = Response to Script Driven Imagery Scale.

In summary, when examining state changes in emotional experience over NFB training in response to trauma/stressful stimuli presentation, we found that the PTSD and healthy control groups demonstrated significant reductions in reliving symptoms, where additionally, the PTSD group demonstrated significant reductions on distress symptoms as measured by the RSDI Scale (see Figure 7).

Specifically, the PTSD group showed a significant main effect of run for the nonparametric ANOVA examining symptoms of reliving (*χ* ^2^(3) = 11.49, *p* = .009). Follow‐up Wilcoxon signed‐rank tests revealed that NFB run 3 had lower scores of reliving than NFB run 1 (*p* = .016). Similarly, the control group demonstrated a significant main effect of run examining symptoms of reliving (*χ* ^2^(3) = 18.24, *p* < .0001). Follow‐up tests revealed that NFB runs 3 (*p =* .008) and 4 (*p =* .010) had significantly lower scores of reliving than NFB run 1. Finally, the PTSD group also displayed a significant main effect of run for symptoms of distress (*χ* ^2^(3) = 13.79, *p =* .003). Follow‐up tests showed that distress symptoms during NFB run 3 (*p =* .010) and 4 (*p =* .013) were significantly lower than NFB run 1. Symptoms of physical reactions, dissociation and numbing did not significantly decrease over NFB runs. Furthermore, as expected, when comparing state symptoms across NFB runs between groups with Mann–Whitney U tests, the PTSD group always demonstrated higher levels of symptoms as compared to the healthy control group, even for symptoms of distress and reliving which decreased significantly over NFB runs for the PTSD group.

### 3.5 Machine learning classification analysis

Supporting differential mechanisms associated with PCC downregulation in PTSD versus healthy individuals (i.e., psychopathological specificity), machine learning algorithms were able to classify participants based on NFB brain activation during the *view* as compared to *regulate* condition during training runs with 80% accuracy (ROC = 0.85, *p* < .001 permutation testing). The class predictive value was 83.33% for the PTSD group and 76.47% for the healthy control group. Additionally, the class accuracy was 71.43% for the PTSD group and 86.67% for the healthy control group. The highest‐ranking ROIs used by the decision function of the machine were bilateral the dlPFC, the bilateral dmPFC, the bilateral vmPFC, and the PCC, which provides converging evidence of our univariate between group results. Here, the MKL models were indeed quite sparse, with nine regions having a nonnull contribution across folds.

## 4 DISCUSSION

In summary, we found that both the PTSD and healthy control groups were able to downregulate their PCC with similar success in terms of average event‐related BOLD response within the NFB target area, which remained stable over NFB training and into the post‐training transfer run. With regard to state changes in emotional experience over NFB training, both the PTSD and healthy control groups demonstrated reduced reliving symptoms in response to trauma/stressful stimuli. Additionally, the PTSD group demonstrated significantly reduced distress symptoms over NFB training and into the post‐training transfer run. Interestingly, PCC NFB training was concomitantly associated with unique within‐group downregulation of activity (*regulate < view*) within the dorsomedial PFC, postcentral gyrus, amygdala/hippocampus, cingulate cortex, and temporal pole/gyri. Interestingly, downregulating the PCC during NFB training was associated with greater activation (*regulate > view)* in the right dlPFC among healthy individuals as compared to those with PTSD. In support of this, increased activation in the right dlPFC during *regulate* as compared to *view* conditions was negatively correlated to PTSD symptom severity scores and difficulties in emotion regulation. Furthermore, stronger activation in the anterior insula and cerebellum (lobule VI/crus I) during *view* as compared to *regulate* conditions was positively associated with PTSD symptoms.

Supporting differential mechanisms associated with PCC downregulation in PTSD versus healthy individuals, machine learning models were able to classify participants based on brain activation during *view* as compared to *regulate* conditions *during NFB training runs* with 80% accuracy. Here, the highest‐ranking ROIs used by the decision function of the machine were the bilateral dlPFC, the bilateral dmPFC, the bilateral vmPFC, and the PCC, which provides converging evidence of our univariate between group results.

### 4.1 PCC neurofeedback decreased reliving and distress symptoms

Within both the PTSD and healthy control groups, the BOLD response within the PCC search volume (NFB target area) was found to be significantly lower during the *regulate* as compared to *view* conditions for all three NFB training runs and the transfer run, where PCC activation during *regulate* did not differ significantly when comparing NFB runs. Direct comparisons of regulation success did not yield significant between group differences, suggesting that PTSD and healthy individuals can similarly gain control over this brain region via rt‐fMRI‐NFB. Further demonstrating the strength of the current results, at the whole‐brain FDR‐corrected level, both groups showed decreased activation within clusters spanning the PCC/precuneus during *regulate* as compared to *view* conditions over NFB‐training. Interestingly, these whole‐brain results extended to the transfer run only for the healthy control group, where additional training sessions/statistical power may be required to observe this transfer run effect in the PTSD group at our conservative statistical threshold (FDR *p* <.05). The PCC and precuneus are major hubs of the DMN, where DMN functional disruptions in PTSD are associated with traumatic/negative autobiographical memories, distorted and dysregulated self‐referential processing, and alterations in social cognition (Bluhm et al., 2009; Daniels et al., 2010; Lanius et al., 2015, 2020; Tursich et al., 2015; Akiki et al., 2017; Fenster et al., 2018; Hinojosa et al., 2019; Frewen et al., 2020; Terpou et al., 2020). Indeed, traumatic imagery tasks in PTSD have been found to induce hyperactivation in the PCC (Awasthi et al., 2020) and in the precuneus (Ramage et al., 2013), where it has been suggested that suboptimal downregulation of the PCC and DMN may underscore difficulties in disengaging from internally focused self‐referential processing (Aupperle et al., 2016). A recent meta‐analysis has also found that both reexperiencing and retrieval of trauma‐related autobiographical memories are associated with enhanced activation within the PCC and other DMN regions among individuals with PTSD (Thome et al., 2019). In line with these findings, we found increased PCC activation during *view* as compared to *neutral* conditions in the current study. Notably, NFB training of PCC downregulation resulted in concomitant decreases in reliving symptoms among those with PTSD and healthy individuals, with additional decreases in distress symptoms in the PTSD group. As expected, between‐group comparisons revealed that reliving and distress symptoms were higher in the PTSD group as compared to the healthy control group. In support of associations between PCC downregulation and reductions in symptoms, longitudinal improvements in PTSD symptoms in response to trauma‐focused cognitive behavioral therapy (CBT) have been found to be associated with decreased PCC activation in youth with PTSD during emotion‐processing tasks (Garrett et al., 2019).

Consistent with the current results, one session of alpha rhythm EEG‐NFB has been shown previously to lead to acute decreases in arousal symptoms among PTSD patients and normalize both DMN and amygdala resting‐state functional connectivity patterns (Kluetsch et al., 2014; Nicholson et al., 2016b). Additionally, during a 20‐week randomized controlled trial of alpha‐based EEG‐NFB in PTSD (Nicholson et al., 2020b), individuals in the experimental group demonstrated clinically meaningful reductions on PTSD severity scores post‐NFB and at 3‐month follow‐up, which were associated with a shift towards normalization of DMN resting‐state functional connectivity.

### 4.2 NFB‐induced whole‐brain regulation and correlations with symptoms

When examining whole‐brain BOLD response during PCC NFB training runs, with respect to *regulate* as compared to *view* conditions, the **PTSD group** showed decreased activity in the bilateral dmPFC, the mid‐cingulate cortex, and the left amygdala/hippocampus. Previously, the presentation of trauma‐versus‐neutral words has been shown to be related to increased activation within the DMN (the PCC and mPFC) and the SN (the PAG and BNST), as compared to decreased activation in the CEN involved in emotion regulation (the dlPFC) when comparing PTSD and healthy controls (Awasthi et al., 2020). In the same study, PTSD symptom severity was positively correlated with neural activation during trauma‐versus‐neutral words within the DMN (the PCC and hippocampus) and the SN (the amygdala and BNST) and negatively correlated with CEN activation (the dlPFC). This is supported by previous research examining PTSD brain correlates during both script‐driven imagery and the recall of trauma‐related autobiographical memories in PTSD (Hopper et al., 2007a; Lanius et al., 2007; Frewen et al., 2011; Mickleborough et al., 2011; Ramage et al., 2013; Liberzon and Abelson, 2016; Fenster et al., 2018; Thome et al., 2019). Interestingly, in the current study, we found evidence to suggest that downregulating the PCC during trauma word presentation resulted in a normalization of this PTSD neural signature, with decreased activation in aforementioned DMN regions involved in self‐related and autobiographical memory processing (bilateral dmPFC and hippocampus) as well as in SN areas involved in emotional arousal, emotion evaluation, salience monitoring and innate fight‐or‐flight defensive responses (amygdala and mid‐cingulate cortex) (Liddell et al., 2005; Etkin et al., 2011; Lanius et al., 2015, 2017; Fitzgerald et al., 2018; Hinojosa et al., 2019). Activation in the dmPFC occurs during self‐related emotion processing and exposure to negative content, and additionally, is involved in evaluating self‐related emotional experience (Fitzgerald et al., 2018). Moreover, the dmPFC has been shown generally to subserve functions related to the appraisal and expression of fear and anxiety (Etkin et al., 2011). Recent meta‐analyses have also reported that the mid‐cingulate cortex is hyperactive in PTSD, with both correlations to PTSD severity and trauma exposure (Hayes et al., 2012; Patel et al., 2012; Hinojosa et al., 2019). Furthermore, several studies have found evidence of enhanced hippocampal engagement during exposure to trauma‐specific images, as well as increased activation during reexperiencing and retrieval of trauma‐related autobiographical memories (Hou et al., 2007; Nilsen et al., 2016; Fitzgerald et al., 2018; Thome et al., 2019). With respect to the amygdala, due to its hyperactivity in close association with symptoms of PTSD and hyperarousal (Hayes et al., 2012; Fenster et al., 2018; Fitzgerald et al., 2018; Henigsberg et al., 2018) and its involvement with the innate alarm system in the salient detection of threat (Liddell et al., 2005; Lanius et al., 2017), this limbic region has been a frequent target of previous rt‐fMRI‐NFB studies in PTSD (Gerin et al., 2016; Nicholson et al., 2016a, 2018; Misaki et al., 2018b, 2019; Zotev et al., 2018; Chiba et al., 2019). Indeed, it has been shown recently that high treatment response among PTSD patients is characterized by less amygdala‐PCC connectivity at rest (Sheynin et al., 2020). Taken together, results from the current study suggest that regulating the PCC not only results in gaining control over other DMN structures (dmPFC, hippocampus) but also results in the downregulation of SN structures (amygdala, mid‐cingulate) that have been shown previously to be hyperactive and tied to PTSD symptoms during trauma‐provocation.

Between‐group comparisons revealed increased right dlPFC activation in the healthy control group relative to the PTSD group during *regulate* conditions as compared to *view* conditions. In support of this, increased activation in the right dlPFC during *regulate* as compared to *view* conditions was negatively correlated to global PTSD symptom severity scores (as well as intrusion, negative alterations in cognitions and mood, and arousal symptom clusters) and difficulties in emotion regulation. In other words, the more dlPFC activation during *regulate* conditions, the less PTSD symptoms and less difficulties in emotion regulation. As mentioned previously, recent studies have shown decreased dlPFC activation in PTSD during the presentation of trauma‐versus‐neutral words as compared to healthy controls (Awasthi et al., 2020), where hypoactivation within this region has been commonly associated with PTSD symptoms (Pitman et al., 2012; Fenster et al., 2018; Fitzgerald et al., 2018; Holmes et al., 2018). Critically, we have shown previously that amygdala downregulation training with rt‐fMRI‐NFB leads to both increased dlPFC activation and increased recruitment of the CEN during emotion induction paradigms (Nicholson et al., 2016a, 2018). Zotev et al. (2018) showed that 3‐sessions of amygdala training with rt‐fMRI‐NFB lead to reductions in PTSD severity that correlated with enhanced functional connectivity between the amygdala and dlPFC. In the same data set, a connectome‐wide investigation revealed that increased resting‐state connectivity between the left dlPFC and the precuneus was correlated with PTSD symptom reductions in hyperarousal after the three NFB training sessions (Misaki et al., 2018a). Indeed, future studies are warranted to investigate if multiple sessions of PCC downregulation with rt‐fMRI‐NFB would also result in increased dlPFC recruitment among individuals with PTSD and if this neural response mediates PTSD symptoms.

In addition to dlPFC activation being negatively correlated with PTSD symptoms, our results also revealed that activation in the anterior insula during *view* as compared to *regulate* conditions was positively associated with global PTSD symptom severity scores (as well as negative alterations in cognitions and mood, and arousal symptom clusters) and difficulties in emotion regulation. Previously, the anterior insula has been shown to be hyperactive in PTSD with positive correlations to symptoms of reexperiencing; in addition, this area also displays aberrant functional connectivity patterns during the resting‐state in PTSD (Hopper et al., 2007b; Nicholson et al., 2016c; Fenster et al., 2018; Harricharan et al., 2019). It has been suggested that increased anterior insula activity may coincide with enhanced salience processing of environmental cues and PTSD symptoms of hypervigilance, hyperarousal, and reexperiencing (Hopper et al., 2007b; Patel et al., 2012; Koch et al., 2016; Akiki et al., 2017). Our recent randomized clinical trial of alpha rhythm EEG‐NFB over 20 weeks also revealed decreased anterior insula connectivity with the salience network after NFB intervention (Nicholson et al., 2020b). In support of these findings, a recent review also suggests that treatment response in PTSD is associated with lower functional activity and connectivity within the anterior insula (Szeszko & Yehuda, 2019). Furthermore, activation in the right cerebellum (lobule VI/crus I) during *view* as compared to *regulate* conditions was positively correlated to global PTSD symptom severity scores (as well as intrusion, and negative alterations in cognitions and mood symptom clusters). Indeed, lobule VI/crus I regions of the posterior cerebellum are dedicated to cognitive and executive functions, working memory, visuospatial functions, and limbic system processing (Stoodley and Schmahmann, 2009) and have been shown previously to be associated with PTSD symptomatology (Rabellino et al., 2018).

Common to both groups was the downregulation of various regions in the postcentral gyri, the temporal gyri, and the temporal pole during *regulate* conditions as compared to *view* conditions. The postcentral gyrus represents the primary somatosensory cortex and plays a crucial role in somatosensory representations linked to the perception of emotional and sensory experience (Kragel & LaBar, 2016; Cao et al., 2018). This is in line with a body of literature that suggests that emotions are represented in the somatosensory system as categorically distinct somatotopic maps associated with unique bodily sensations (Nummenmaa et al., 2014). Critically, the postcentral gyrus, along with the superior, mid, and inferior temporal gyri, has been shown to be implicated in the innate alarm system involved in the ultrafast salient detection of danger (Liddell et al., 2005; Lanius et al., 2017). In relation, the temporal pole is a paralimbic region that is highly interconnected with the amygdala and orbitofrontal cortex and is involved in both sensory and limbic processing underlying emotional states (i.e., affective processing) (Olson et al., 2007). Previously, we have shown that the temporal pole is hyperconnected to the salience network at rest among PTSD patients as compared to healthy individuals (Nicholson et al., 2020b), where the temporal pole has also been shown to be activated during trauma‐related autobiographical memory recall and correlated to PTSD symptoms of reexperiencing (Frewen et al., 2011; Thome et al., 2019). Interestingly, the temporoparietal junction, which includes the posterior superior temporal gyrus, is critical for multisensory integration, bodily self‐consciousness, and embodiment (Arzy et al., 2006; Blanke, 2012; Igelström et al., 2015). Additionally, the superior, inferior, and mid‐temporal gyri have been shown to display increased activation in response to trauma‐related stimuli and have been correlated to PTSD symptoms of avoidance and dissociation (Lanius et al., 2002; Hopper et al., 2007b; Nilsen et al., 2016). Indeed, it has been shown recently that PCC and middle temporal gyrus connectivity negatively correlate with PTSD severity and reexperiencing (Sheynin et al., 2020). Although speculative, results from the current study suggest that while downregulating the PCC with NFB during the presentation of trauma stimuli, individuals are also gaining control over the sensory‐experience of their trauma‐related emotions that are mapped within the somatosensory system. Furthermore, our results also suggest that PCC downregulation results in concomitantly gaining control over temporal lobe regions highly associated with PTSD symptoms, in addition to embodiment, and sensory/limbic processing of emotional states.

Finally, during the transfer run, we found nonsignificant whole‐brain activation differences between *regulate* and *view* conditions within the PTSD group and when comparing these conditions between the PTSD and healthy control groups. Nevertheless, within the transfer run, the healthy control group displayed decreased precuneus/PCC and right postcentral gyrus activation during the transfer run for the *regulate* as compared to *view* conditions. This may represent more sustained downregulation effects and optimized NFB learning among healthy controls, as these areas are also shown to be downregulated during the NFB training. Future studies are warranted to investigate if multiple sessions of PCC downregulation with rt‐fMRI‐NFB would also result in these sustained transfer run effects within the PTSD group.

### 4.3 Future directions and limitations

Moving forward, future investigations should examine the combined effects of multiple sessions of NFB‐training with respect to PCC downregulation, as well as collect follow‐up measures in order to examine sustained effects of this intervention. Functional and effective connectivity analyses of the PCC are also warranted. Furthermore, future studies should examine unique responses to treatment among heterogeneous presentations of PTSD, including the dissociative subtype and complex PTSD diagnoses. In the current study, the influence of past psychotherapy and psychotropic medication on neurofeedback training success was not evaluated. Future studies are needed to examine both the interactive effect of neurofeedback and psychotherapy/psychotropic medication, as well as the use of neurofeedback as an adjunctive treatment for PTSD. Lastly, ideally powered randomized controlled trials with comparisons to sham‐NFB and mental rehearsal conditions are also required (Sorger et al., 2019).

## 5 CONCLUSIONS

In summary, we found that both the PTSD and healthy control groups were able to downregulate the PCC with similar success over NFB training and in the transfer run. Indeed, both the PTSD and healthy control groups demonstrated reduced reliving symptoms in response to trauma/stressful stimuli, where the PTSD group additionally demonstrated significantly reduced distress symptoms over NFB training. Here, PCC downregulation was associated with unique within‐group *decreases* in activation within the dmPFC, postcentral gyrus, amygdala/hippocampus, cingulate cortex, and temporal pole/middle and superior temporal gyri. By contrast, downregulation was associated with *increased* activation in the right dlPFC among healthy controls as compared to PTSD. During PCC downregulation, right dlPFC activation was negatively correlated to PTSD symptom severity scores and difficulties in emotion regulation. Moreover, anterior insula and cerebellum (lobule VI/crus I) activation was positively correlated to PTSD symptoms. Finally, machine learning algorithms were able to classify participants based on brain activation during NFB training with 80% accuracy. Importantly, this is the first study to investigate PCC downregulation with real‐time fMRI NFB in PTSD. Taken together, our results reveal acute decreases in symptoms during PCC NFB training and provide converging evidence for alpha EEG‐NFB targeting brain networks linked to the PCC. Future clinical trials of rt‐fMRI‐NFB investigating PCC downregulation in PTSD are warranted to leverage the effects of multiple training sessions.

### PEER REVIEW

The peer review history for this article is available at https://publons.com/publon/10.1002/brb3.2441

## Supporting information

Supporting Information

Supporting Information

## Data Availability

The data that support the findings of this study are available from the corresponding author upon reasonable request.
