# Supplementary material for: Differential mechanisms of posterior cingulate cortex downregulation and symptom decreases in posttraumatic stress disorder and healthy individuals using real‐time fMRI neurofeedback
Source: Brain Behav. 2021 Dec 18;12(1):e2441. doi: 10.1002/brb3.2441 (PMC8785646; doi:10.1002/brb3.2441)
Supplement: Supplementary file 1 — Supporting Information [file BRB3-12-e2441-s001.docx]

**Table s1**

*History of Trauma Exposure Based on the Life Events Checklist for DSM-5 (LEC-5)*

| **Event** | | **PTSD Group** | **Healthy Control Group** |
| --- | --- | --- | --- |
| 1. | Natural disaster (e.g. flood, hurricane, tornado, earthquake) | *n=*7 | *n=*2 |
| 2. | Fire or explosion | *n=*6 | *n=*1 |
| 3. | Transportation accident (e.g. car accident, boat accident, train wreck, plane crash) | *n=*10 | *n=*8 |
| 4. | Serious accident at work, home, or during recreational activity | *n=*8 | *n=*1 |
| 5. | Exposure to toxic substance (e.g. dangerous chemicals, radiation) | *n=*8 | *n=*1 |
| 6. | Physical assault (e.g. physical abuse, being attacked, hit, slapped, kicked, beaten up) | *n=*13 | *n=*4 |
| 7. | Assault with a weapon (e.g. being shot, stabbed, threatened with a knife, gun, bomb) | *n=*8 | *n=*2 |
| 8. | Sexual assault (rape, attempted rape, made to perform any type of sexual act through force or threat of harm) | *n=*9 | *n=*1 |
| 9. | Other unwanted or uncomfortable sexual experience (e.g. inappropriate touching) | *n=*9 | *n=*3 |
| 10. | Combat or exposure to a war-zone (in the military or as a civilian) | *n=*5 | *n=*1 |
| 11. | Captivity (e.g. being kidnapped, abducted, held hostage, prisoner of war) | *n=*1 | *n=*0 |
| 12. | Life-threatening illness or injury | *n=*3 | *n=*1 |
| 13. | Severe human suffering | *n=*4 | *n=*0 |
| 14. | Witnessing sudden violent death (e.g. homicide, suicide) | *n=*5 | *n=*0 |
| 15. | Witnessing sudden accidental death | *n=*5 | *n=*0 |
| 16. | Serious injury, harm, or death you caused to someone else | *n=*6 | *n=*1 |
| 17. | Neglect by a caregiver (e.g. not getting the emotional or physical things you needed growing up, such as love, support, food, clean clothes) | *n=*11 | *n=*3 |
| 18. | Any other very stressful event or experience | *n=*1 | *n=*3 |

**Figure s1**

**
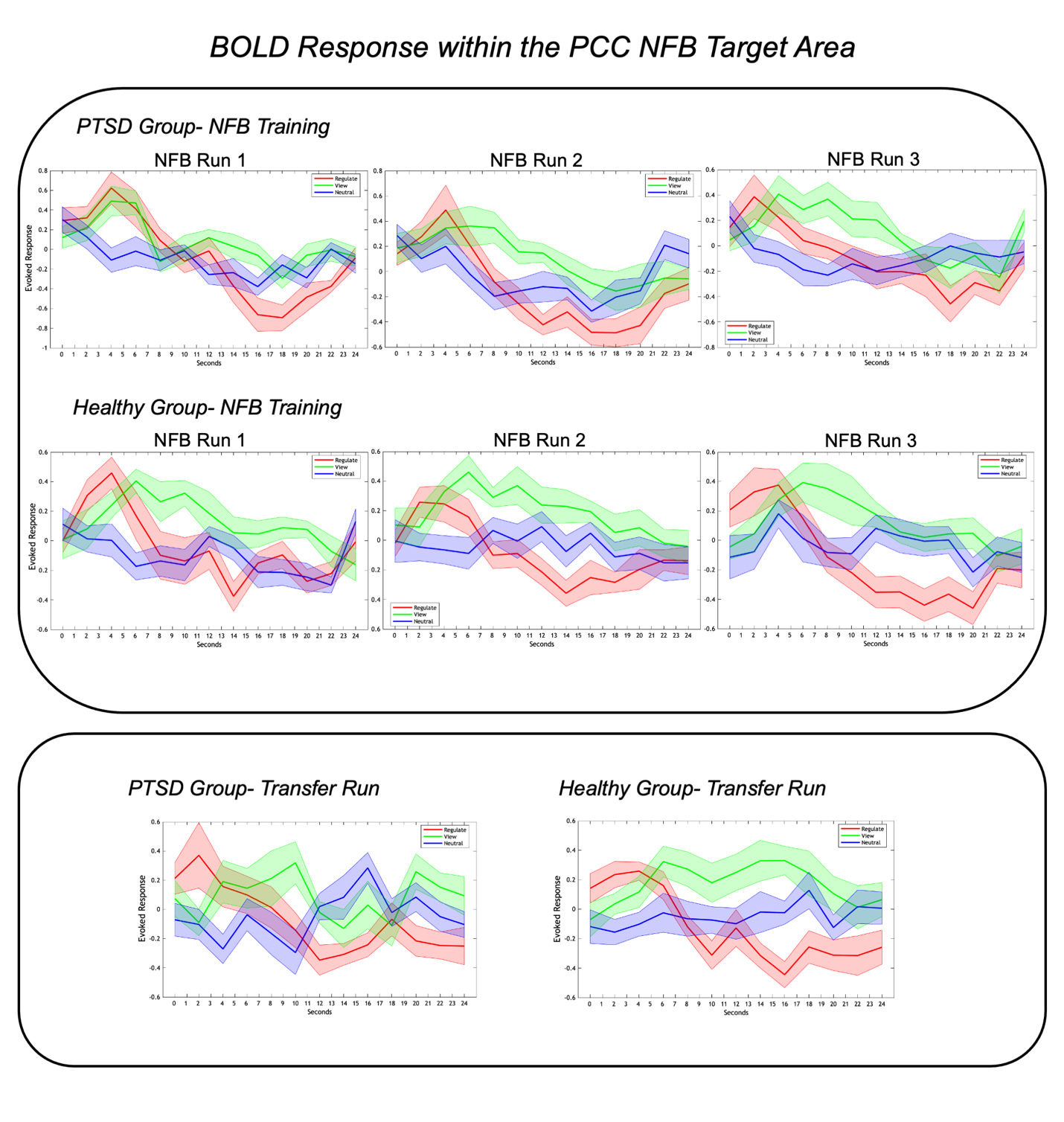
**

**Figure s1 Caption**: A) Event-related BOLD response in the NFB target area (PCC), during the three training runs in the PTSD and healthy control groups. The red lines indicate PCC activation during the *regulate* condition, where the goal was to decrease activation while viewing trauma/stressful words. The green lines indicate PCC activation during the *view* condition, where participants were not attempting to decrease activation while viewing trauma/stressful words. The blue line indicates PCC activation during the *neutral* condition, where participants were not attempting to decrease activation while viewing neutral words. B) Event-related BOLD response in the target area (PCC) during the transfer run when neurofeedback was not provided. Taken together, this demonstrates that both groups were able to gain control over downregulating their PCC with similar success. The x-axis of the graphs indicate time over the 24s conditions; the y-axis indicates the event-related BOLD response (peri-stimulus time histogram) in the target area. Shaded areas of red, green, and blue indicate standard error of the mean.

Abbreviations: PCC= posterior cingulate cortex, NFB= neurofeedback.

**Table s2**

*Direct Comparison of View versus Neutral Conditions Across Participants*

| **Contrast** | **Brain Region** | **H** | **Cluster Size** |  | **MNI Coordinate** |  | **t Stat.** | **Z Score** | ***p-FDR* Cluster Level** |
| --- | --- | --- | --- | --- | --- | --- | --- | --- | --- |
|  |  |  |  | **x** | **y** | **z** |  |  |  |
| View > Neutral | Anterior Insula/frontal operculum | L | 107 | -40 | 26 | 2 | 8.91 | 6.09 | <.020 |
|  | Cerebellum Crus I/II | R | 2458 | 24 | -76 | -38 | 8.18 | 5.80 | <.0001 |
|  | PCC/precuneus | L | 1431 | -16 | -48 | 28 | 7.05 | 5.30 | <.0001 |
|  | Cerebellum Crus I/II | L | 108 | -32 | -76 | -36 | 5.95 | 4.74 | <.020 |
|  | dmPFC | L | 115 | -6 | 48 | 28 | 4.89 | 4.12 | <.020 |
|  | Angular Gyrus | L | 107 | -46 | -68 | 32 | 4.63 | 3.95 | <.020 |
|  | Cerebellum Lobule VI/Crus I | L | 135 | -36 | -60 | -30 | 4.62 | 3.95 | <.020 |
| Neutral > View | *ns* |  |  |  |  |  |  |  |  |

Results of the direct follow-up comparisons for all NFB runs, within all participants, for the *view* as compared to *neutral* conditions, evaluated at the FDR-cluster corrected threshold for multiple comparisons (*p*<.05, k=10).
