# Supplementary figures and images for: Differential mechanisms of posterior cingulate cortex downregulation and symptom decreases in posttraumatic stress disorder and healthy individuals using real‐time fMRI neurofeedback
Source: Brain Behav. 2021 Dec 18;12(1):e2441. doi: 10.1002/brb3.2441 (PMC8785646; doi:10.1002/brb3.2441)

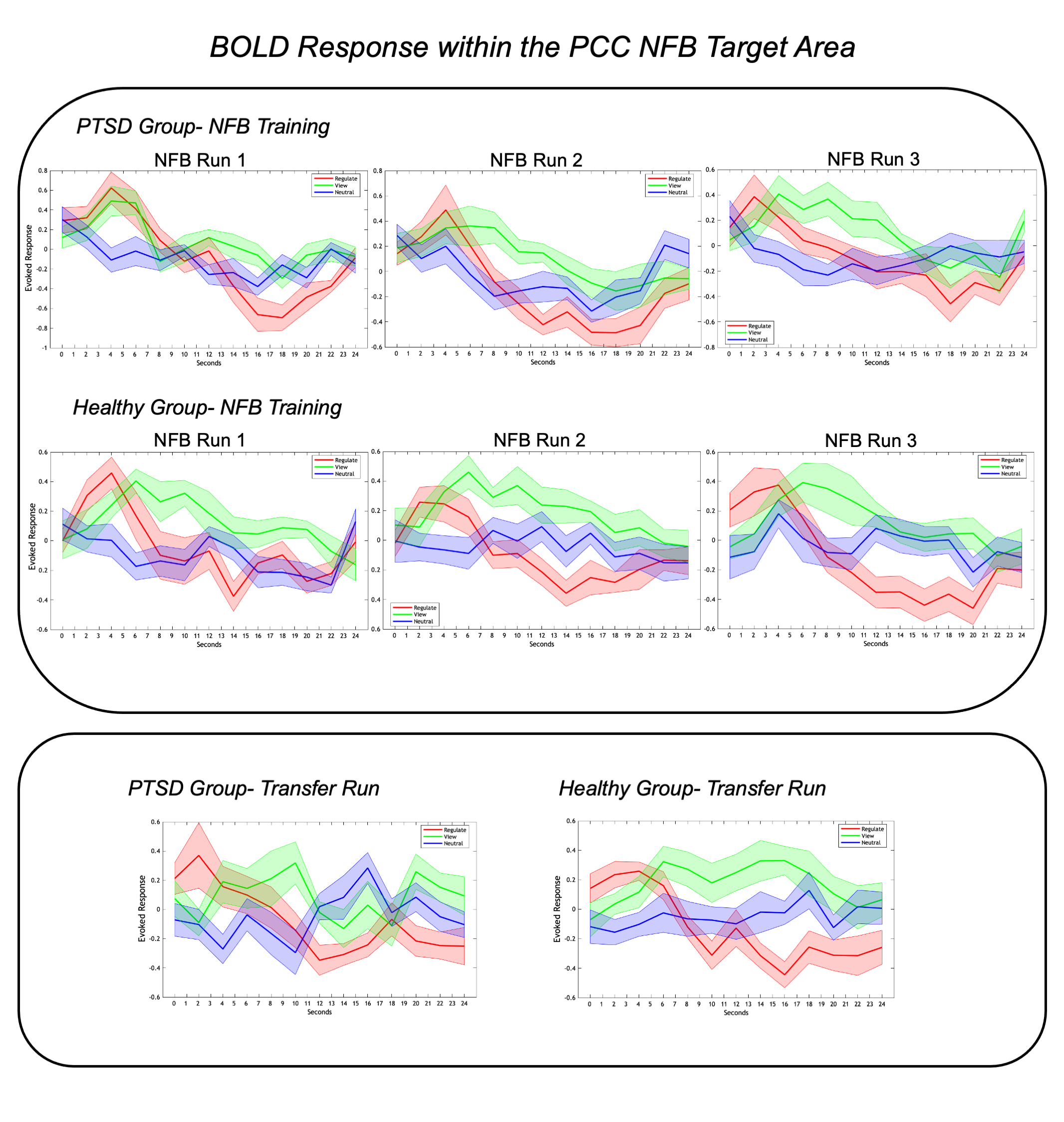

Supplement: Supplementary file 2 — Supporting Information [file BRB3-12-e2441-s002.tiff]
